# More than flowers: Habitat type, floral resources, and landscape context shape pollinator communities in villages

**DOI:** 10.1002/eap.70190

**Published:** 2026-02-24

**Authors:** Sonja Schulze, Fabienne Maihoff, Jie Zhang, Daniela Kessner‐Beierlein, Alicia Bender, Annika Schöninger, Andrea Holzschuh, Ingolf Steffan‐Dewenter

**Affiliations:** ^1^ Department of Animal Ecology and Tropical Biology, Biocenter University Würzburg Würzburg Germany

**Keywords:** biodiversity conservation, fallows, flower preferences, gardens, graveyards, landscape, native plants, ornamental plants, pollinators, public green space, settlements, urban ecology

## Abstract

Wild pollinator diversity has been widely studied in agricultural habitats and increasingly also in cities, but the value of small settlements like villages in rural areas for pollinators is mainly unknown. Public green spaces and village gardens could serve as refuges from agricultural intensification and habitat loss. Moreover, semi‐natural habitats in the surrounding landscape may influence pollinator communities within villages. Here, we asked how suitable different village habitats are for wild pollinators and how this relates to floral resources and landscape context. We recorded solitary bees, bumble bees, hoverflies, honey bees, and flowering plants in five habitat types—cemeteries, fallows, farmhouse gardens, green areas, and house gardens—across 40 villages in Bavaria, Germany (200 plots in total). We recorded 208 wild bee species and 56 hoverfly species representing approximately 40% and 14% of the Bavarian fauna, respectively, along with 1258 flowering plant species. Generally, pollinator richness and abundance increased with floral species richness and cover. The proportion of semi‐natural habitats surrounding villages at larger spatial scales was positively associated with solitary bee richness and influenced bumble bee abundance, highlighting the importance of landscape context. Based on predictions from floral resources, solitary bee richness in green areas and bumble bee richness in fallows exceeded expectations, whereas cemeteries were less species‐rich. This suggests that factors beyond flower richness and abundance, such as nesting opportunities and the composition of preferred flower species, play important roles. Using 38,620 recordings of flower visits and respective flower abundance, we compiled a list of plant genera that were most visited, most preferred (corrected for plant abundance), or non‐preferential (corrected for plant abundance) for the pollinator groups. The list serves as a decision‐making tool for local stakeholders to ensure the most effective pollinator promotion within villages. Our results suggest that measures enhancing flower resources alone will not result in the best possible increase in pollinators in villages but should be accompanied by actions that enhance nesting sites in local habitats for a broad spectrum of pollinators. In conclusion, villages hold a substantial, yet underexploited, potential for pollinator conservation, achievable through targeted management and public engagement.

## INTRODUCTION

Most research on pollinators addresses the effects of habitat fragmentation or land‐use intensification in near‐ or semi‐natural habitats and agricultural landscapes, or, more recently, the value of urban habitats (Moquet et al., 2018; Theodorou et al., 2020; Winfree et al., 2009). Less attention, however, has been given to small‐scale forms of human development embedded within agricultural landscapes, such as villages and other rural settlements, and thus knowledge about biodiversity in settlement areas remains biased towards large cities (Kendal et al., 2020, González, 2020, but see Batáry et al., 2025). Village settlements, while less extensive and impervious than cities, encompass structurally diverse habitats that do not occur in intensively managed farmland. These include differently managed private and public spaces, with a variety of vegetation types, which can create distinct floral resources and increase habitat heterogeneity. Because of their location within predominantly agricultural landscapes, such settlements may play a role as pollinator refugia (Nunes et al., 2024). The proximity of village habitats to surrounding semi‐natural areas creates opportunities for habitat connectivity and access to additional food and nesting resources within pollinator foraging ranges (Batáry et al., 2025; Biegerl et al., 2025; Brückmann et al., 2010; Kennedy et al., 2013; Marini et al., 2014). Settlement areas can provide higher flower richness compared to agricultural areas (Ganuza et al., 2022; Udy et al., 2020), which is mostly linked to the presence of non‐native species in private gardens and public green areas, and the intention of private gardeners to cultivate an expanded variety (Knapp & Wittig, 2012; Pysek, 1998). In this sense, they share similarities with urban areas but remain more closely tied to surrounding landscapes, creating a distinct ecological context shaped by the interplay of human management choices and surrounding semi‐natural habitats (González, 2020).

Despite their potential importance, the extent to which small rural settlements can mitigate the drivers of pollinator decline—such as intensive land use, habitat loss, and the scarcity of floral resources and nesting sites—remains uncertain (Potts et al., 2010; Vanbergen and Initiative, 2013). Research is still limited on whether the interplay between habitat, floral resources in settlements, and connectivity to semi‐natural habitats enables these areas to act as pollinator refugia (Knapp et al., 2021; Nunes et al., 2024).

Typical village habitats are cemeteries, fallows, farmhouse gardens, public green areas, and house gardens. All these habitats have the potential to cover both needs of wild pollinators: food and nesting resources. The richness of plant species in villages is comparable to those in cities and exceeds those within agricultural habitats (Udy et al., 2020), which might lead to a high richness of pollinators in villages as documented for some cities (Banaszak‐Cibicka et al., 2018; Fetridge et al., 2008). Pollinator groups such as solitary bees, bumble bees, hoverflies, and honey bees are expected to react differently to management practices, habitat modification, and human choice of plant species (Dylewski et al., 2020), including the contrast in flower availability and quality between ornamental, human‐introduced, and native plants (Altman et al., 2022; Hall et al., 2017). Moreover, the role of different habitat types in supporting pollinators may depend not only on local management but also on the proximity of semi‐natural habitats, and hence on the availability of food and nesting resources in the surrounding landscape (Holzschuh et al., 2013). In addition, flower resource availability and quality, and thus pollinator response, may vary depending on habitat type. This makes it crucial to understand the value of different habitat types and preferences for flowering plant species by different pollinator groups to efficiently promote wild pollinators in human settlements.

Notably, understanding the preferences of different pollinator groups for specific plant genera can be directly implemented in management recommendations for private gardens and village communities. However, as highlighted by Garbuzov and Ratnieks (2014), many of the currently available lists lack transparency about how plant attractiveness to pollinators was assessed, and in some even include non‐attractive plants mistakenly labeled as pollinator‐friendly. In contrast, Kuppler et al. (2023) provide a methodologically transparent evaluation; however, their focus is primarily on seed mixtures for use in an agricultural context rather than on the range of floral resources, shaped by human choice, within village habitat types.

To reveal the value of different village habitats for species‐rich and abundant pollinator communities, we aimed to answer the following research questions: (1) How do village habitats differ in the abundance and richness of floral resources and pollinators? (2) Are pollinator richness and abundance modulated by landscape context? (3) Do habitat types differ in their dependence of source habitats in the surrounding landscape? (4) Do habitat types affect pollinator abundance and richness independent from floral resources? and (5) Which plant genera are preferred by different pollinator groups?

## MATERIALS AND METHODS

### Study area

**FIGURE 1:**
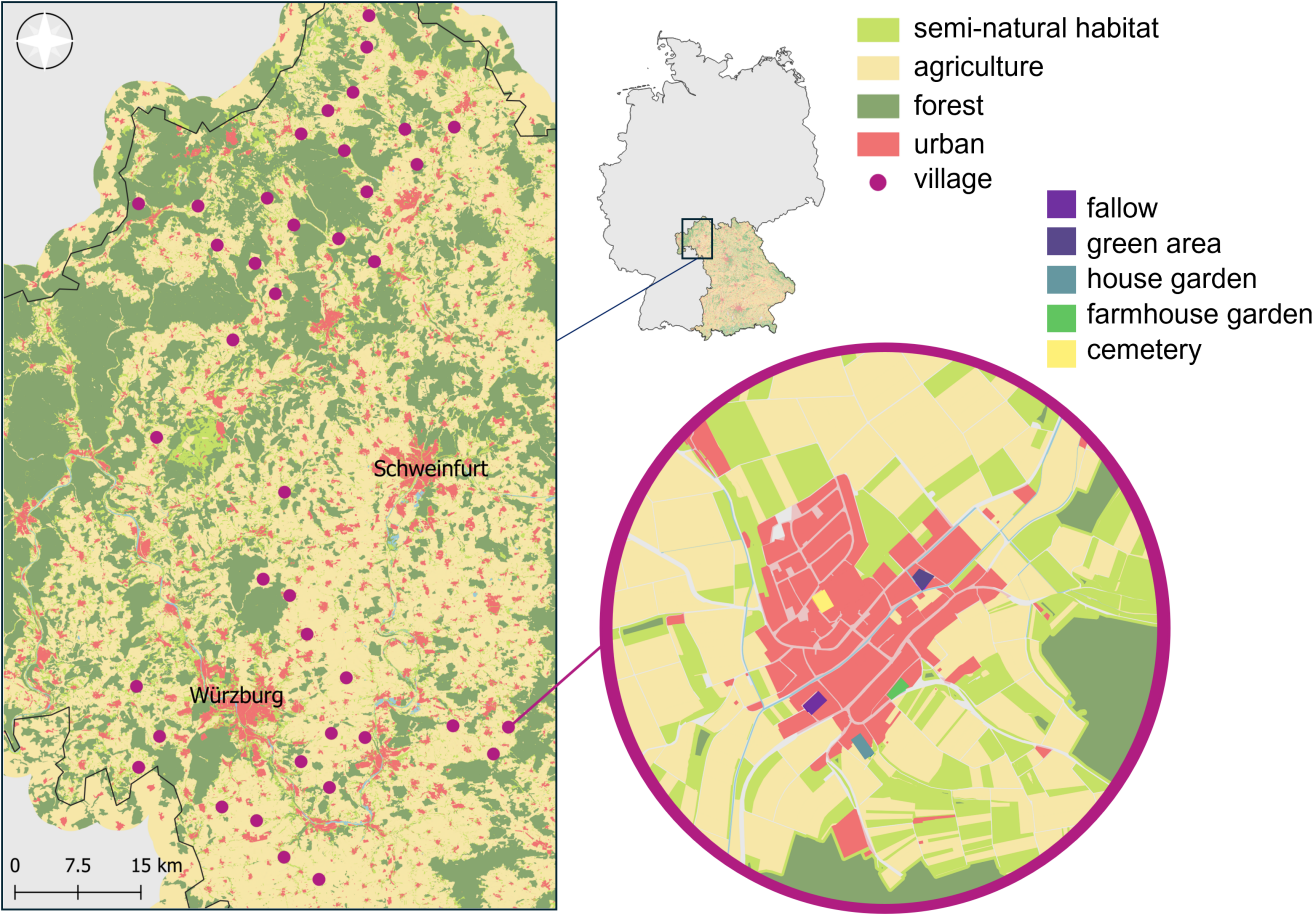
Map of 40 villages taking part in the project. The map on the top shows the position of our study region within Bavaria (Germany). In each village, five habitats, fallow, green area, house garden, farmhouse garden, and cemetery, were studied.

The study was conducted from April to August 2020 on 200 plots in 40 villages in Northern Bavaria, Germany (Figure 1). To select suitable villages, we chose two regions (Rhön and Mainfranken around Würzburg) to cover a gradient in the composition of surrounding landscapes and climatic conditions. Within the two regions, we focused on villages with 800–3000 residents and a minimum distance of six kilometers between each other. The selected villages had a mean area of 51.9 ha ± SD 23.2 ha (largest area = 115.7 ha and smallest area = 16.9 ha).

Within each village, we chose five plots of the following habitat types: fallows, public or private green areas (subsequently “green areas”), farmhouse gardens, residential gardens (subsequently “house gardens”), and cemeteries (Appendix S1: Figure S1). Selection took place in early spring, with permission obtained from either private owners or municipal stakeholders before the flowering season. Consequently, flower cover and richness were not evaluated at this stage; rather, management and structural diversity served as the main criteria. The habitat type “fallows” ranged from unmanaged areas to grasslands that were mown only once or twice a year. In contrast, green areas were mown or mulched at least three times per year, in most cases, even more frequently, resulting in continuously short vegetation. In farmhouse gardens, in contrast to house gardens, the main focus was on production of fruits and vegetables for personal use. House gardens, on the other hand, were predominantly managed for recreation, based on the owners' aesthetic preferences. Given that personal preferences for garden “tidiness” can vary greatly, we covered the full range from neatly pruned and carefully arranged spaces to more naturalistic designs with minimal intervention. The level of tidiness can reflect the owner's preferences for order versus a more spontaneous, wild aesthetic. The majority of plots had an area of at least 500 m^2^, except for 23 plots with an area ranging from 200 to 450 m^2^.

### Pollinator sampling

We conducted five surveys on every plot between April and August 2020, where we recorded the pollinator groups’ “solitary bees” (wild bees excluding the genus *Bombus* but including the partly eusocial genera *Halictus* and *Lasioglossum*), “bumble bees,” “hoverflies,” and “honey bees.” We used transect walks, where we divided each plot into 4 × 125 m^2^ transects, which were sampled for 5 min. Each habitat was therefore surveyed for 20 min (survey time per month). During the handling time of individuals, the timer was stopped. Where plots were larger than 500 m^2^, transects were chosen to reflect the different microhabitats within the plot. When plots were smaller than 500 m^2^, we used the whole area for the transects. Species that could not be identified in the field were taken to the lab for identification. Transect walks took place between 8 AM and 6 PM, as long as certain conditions were met: Temperatures above 15°C in the sun, no rain, and no or only low wind strength (Beaufort‐Scale 0–3). Moreover, when a species was seen visiting a flower, the visited plant species was noted as well. Pollinator abundance and richness were pooled over the four transects and the five surveys for each plot. Pollinator richness was cumulatively pooled, and abundance was summed.

Parallel to the pollinator surveys, we recorded flower richness and flower cover. Flower richness was assessed by documenting each flowering plant species observed during transect walks, separately for each sampling round and transect. Flower cover within the transect was estimated in m^2^ by counting the number of flowers or flower units (e.g., in Asteraceae the flower head) of each plant species per transect and multiplying this by the species' mean flower size. Mean flower size was determined by measuring one flower or flower unit per species detected per transect and averaging all measurements across transects and surveys for each plant species. We cumulated the data for flower richness and summed up the data for flower cover over the transects and surveys for each plot. Moreover, we used the FloraWeb database (Bundesamt für Naturschutz, 2023) to classify plant species as either native or ornamental plant species (“floral status”: native vs. ornamental). Species listed in FloraWeb as either native (= archaeophyte) or established (= since 1492 established in Central Europe) were classified as native. All other species, or those not listed in FloraWeb, were classified as ornamental. This ornamental category also included some heavily cultivated and modified native plants—such as double‐petaled varieties—reflecting species were valued primarily for their aesthetic, human‐introduced role rather than for their naturally established presence. Consequently, vegetable crops and kitchen herbs that are not native to Central Europe were also classified as ornamental. Based on these data, we calculated the flower cover and richness separately for each floral status (native vs. ornamental) and the proportion of ornamentals per plot. For this calculation, we considered only plants identified to the species level, as some genera, such as *Euphorbia*, include both native and ornamental species. This led to the exclusion of 9% of the flower richness and 12% of the flower cover in the calculation of native versus ornamental cover and richness. We identified the most visited plant genera for each pollinator group based on visitation observations over the whole season while taking into account the abundance of flowering plant genera. The exact method is described in the statistical analysis. Trees were not taken into account in this analysis.

### Landscape context

The landscape context, quantified as the proportion of semi‐natural habitats surrounding the study sites (measured from the edge of the study site), was assessed using ArcGIS Pro 2.7.2 (ESRI) with modified digital thematic maps (combining data from Biotopkartierung Bayern, 2021; Integrated Administration and Control System, 2023; CORINE Land Cover, 2023). Semi‐natural habitat comprised (1) orchard meadows, calcareous grasslands, and extensively managed grasslands, (2) forest edges, represented by a 10‐m buffer zone of forests, and (3) fallow stripes and fallow land, and hedges. The proportion of semi‐natural habitat was then calculated within radii of 250, 500, 750, 1000, 1500, 2000, 2500, and 3000 m. Regional climatic differences were addressed on the village level, by the 30‐year multiannual mean air temperature from 1981 to 2010 (Deutscher Wetterdienst, 2020).

### Statistical analysis

All statistical analyses were performed with R version 4.3.1 (R Core Team, 2023).

We used generalized linear mixed models from the package “glmmTMB” version 1.1.7 (Brooks et al., 2017) to analyze whether the fixed factor habitat type had effects on flower richness and the log‐transformed flower cover with the random factor “village” and a Gaussian error distribution. To reduce the influence of extreme values and improve model robustness, flower cover was log‐transformed. In a second step, we tested for habitat‐related differences in flower cover and species richness, analyzing native and ornamental plants separately within each plot (i.e., two values per plot). Both response variables were modeled independently using the same structure, with random intercepts for village and for plots nested within village, specified as (1 | village/plot). We calculated pairwise comparisons using Estimated Marginal Means (“emmeans” or Least Squares Means) with the package “emmeans” version 1.8.7 (Lenth et al., 2023) to determine differences among habitat types in total, native, and ornamental flower richness and in total, native, and ornamental flower cover (Appendix S1: Table S1).

To investigate how local and landscape‐level factors influence pollinator abundance and richness, we constructed a set of generalized linear mixed‐effects models (GLMMs) using the glmmTMB package version 1.1.7 (Brooks et al., 2017). Response variables included the abundance and species richness of the four pollinator groups: solitary bees, bumble bees, honey bees, and hoverflies. Each model included the following fixed effects: habitat type (categorical), log‐transformed flower cover, plant alpha diversity, percentage cover of semi‐natural habitat (SNH) at multiple spatial scales (250–3000 m radius), and multi‐annual mean temperature as covariate to account for temperature‐driven diversity patterns (Brown et al., 2004). Village identity was included as a random intercept. Flower cover was log‐transformed prior to analysis to reduce the influence of extreme values (i.e., right‐skewed distribution) and to limit potential overinterpretation of outliers. Log transformation also helped mitigate multicollinearity with other predictors and improved model interpretability. All continuous predictors were standardized (mean = 0, SD = 1) prior to modeling to facilitate interpretation and reduce multicollinearity (Grueber et al., 2011). With this, the variance inflation factor never exceeded 5, indicating that multicollinearity was not a concern. We used a negative binomial (for hoverfly abundance and solitary bee richness), or a Conway–Maxwell–Poisson distribution when negative binomial models were over‐ or under‐dispersed.

To evaluate the role of interactions between local and landscape predictors across different landscape scales, we fitted four candidate models for each response variable and SNH scale:

1. Baseline model: included all fixed effects but no interaction terms.
2. Interaction model 1: included an interaction between habitat type and floral resource availability to test for habitat‐dependent responses of abundance and richness to flower cover.
3. Interaction model 2: included an interaction between habitat type and SNH cover to test for habitat‐dependent responses of abundance and richness to semi‐natural habitat.
4. Full interaction model: included both interaction terms.

We compared these models using two complementary approaches (Johnson & Omland, 2004; Zuur et al., 2009):

1. Non‐nested model comparison was based on the small‐sample corrected Akaike information criterion (AICc). For each response × SNH combination, we extracted AICc values, marginal *R* ^2^, and conditional *R* ^2^ using the MuMIn and performance packages. The model with the lowest AICc was considered the best‐fitting model, and models within ΔAICc < 2 were interpreted as having equivalent support.
2. Nested model comparison was performed using likelihood ratio tests (LRTs) to evaluate whether the inclusion of interaction terms significantly improved model fit.

To visualize the models, we calculated predictions for each of the response variables with “ggemmeans” from the package “ggeffects” (Lüdecke et al., 2023), while holding the non‐focal value constant at its mean. To assess model diagnostics, we used the package “DHARMa” (Hartig, 2022) and “performance” (Lüdecke et al., 2021).

To assess the effects of ornamental and native plants on pollinator species richness and abundance, we constructed separate models for each response variable (abundance and richness) per pollinator group. In these models, we included the proportion of ornamental plant cover relative to native plant cover as a fixed effect, which allowed us to examine the relative importance of ornamental plants compared to native plants on pollinator communities. Additional fixed effects included total flower cover, total flower richness, multi‐annual mean temperature, and the proportion of semi‐natural habitat within a defined radius, based on the best model structure identified previously. Village and habitat were incorporated as random effects. This approach enabled us to disentangle the relative impacts of ornamental and native plants on pollinator communities in a realistic, context‐dependent manner, as these plant types often coexist on the same sites. Using the “tidy” function from the “broom.mixed” package (Bolker & Robinson, 2024), we calculated the estimated coefficients for each model.

To assess the attractiveness of plants, that is, if plant genera were visited more or less frequently than expected based on their abundance (m^2^ flower cover), we used the package “econullnetr” version 0.2.1 (Vaughan et al., 2018). This package applies a null modeling approach to test for non‐random resource use by consumers and is particularly useful for identifying over‐ or under‐utilized resources in ecological networks. In our case, the four pollinator groups were used as consumers and the plant genera were used as resources. As suggested by Vaughan et al. (2018), we replaced the zero abundance of a plant genus with the very small amount of 0.00001 m^2^ flower cover in case we recorded an interaction between pollinator and the respective plant genus but missed to record the plant genus in the plant abundance data. Double‐petaled flowers, known to offer little or no nectar or pollen, were treated separately due to their limited value to pollinators. A total of 25 such genera were labeled as *genus*_dp and analyzed as distinct from their open‐flowered counterparts, despite belonging to the same genus.

We ran a null model based on the observed interaction data and independent estimates of the flower abundance with 1000 iterations. We applied the *test_interactions* function of “econullnetr” to compare the observed interaction strengths to the estimates of the null model to check if a genus was visited significantly more often than expected (stronger), significantly less often visited than expected (weaker), or consistent with the null model to check for pollinator preferences. We identified for each pollinator group the 15 most visited plant genera (sum of visits per plant genus during the study period), the 15 most preferred plant genera, and the 15 non‐preferential plant genera per pollinator group (both taking flower cover into account). To identify the most and least preferred plant genera, we selected the genera with the highest and the lowest standardized effect size, respectively. The standardized effect size was calculated as the difference between observed and expected link strength (difference in the total number of interactions summed across the individual pollinator group and mean interactions across the iterations of the null model, respectively), divided by the standard deviation of the link strength across the iterations of the null model (Vaughan et al., 2018). To prevent a display of single visitation events on most preferred genera with very low flower cover, we applied a threshold of at least five expected visits.

All graphs were produced with “ggplot2” version 3.4.2 (Wickham et al., 2023).

## RESULTS

In total, we recorded 22,012 solitary bees, 10,325 bumble bees, 12,295 hoverflies, and 10,930 honey bees. We found 192 solitary bee species, 16 bumble bee species, and 56 hoverfly species; 22.5% of the 208 wild bee species found are listed as endangered and another 12.5% as vulnerable (Voith et al., 2021). Moreover, we recorded 1258 flowering plant species within 564 genera (species lists for pollinators and plants can be found in Maihoff & Schulze, 2025).

### Effects of habitat type on floral resources

**FIGURE 2:**
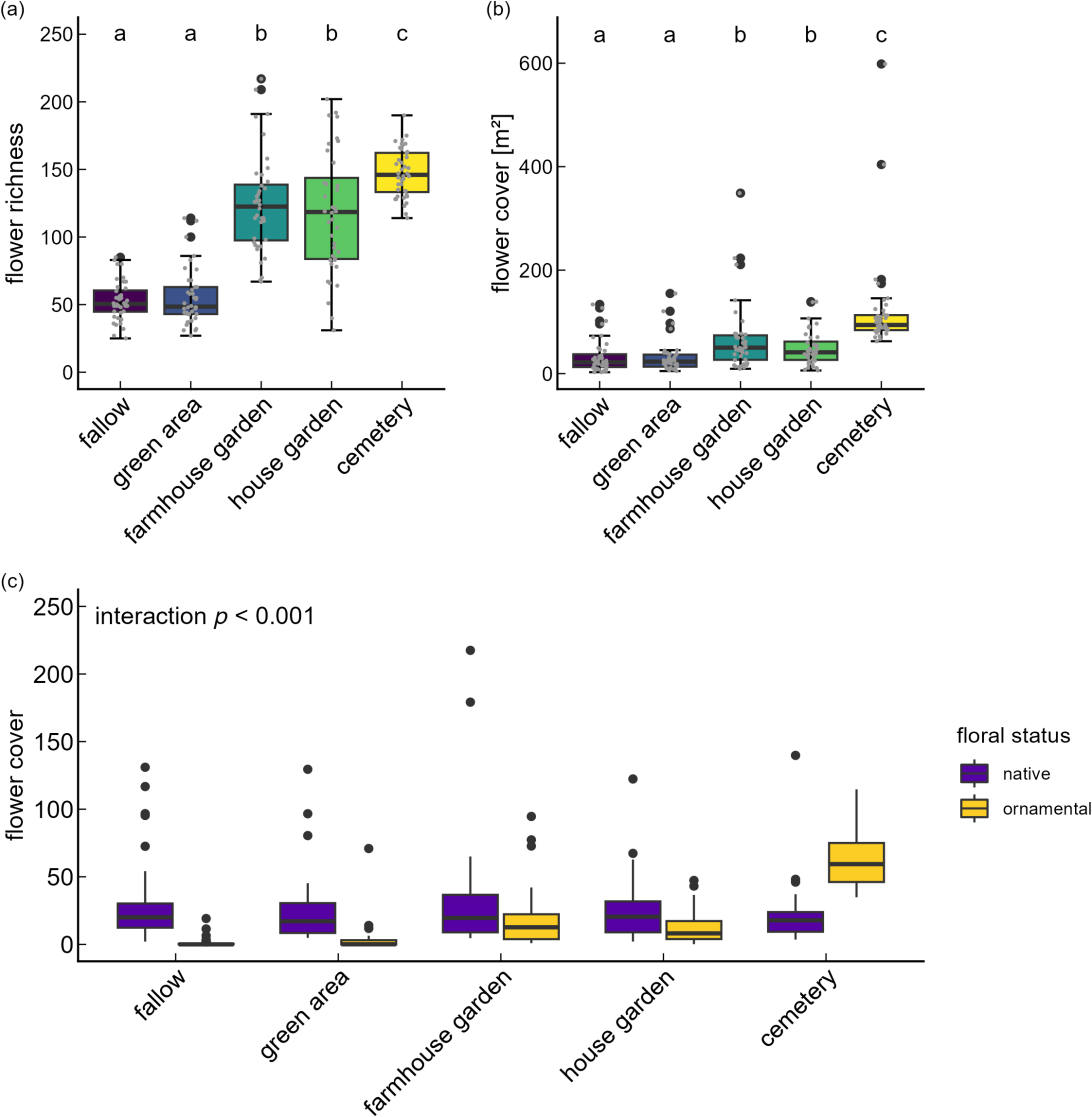
Effect of habitat type on (a) flower richness and (b) flower cover. Points indicate pooled flower richness or flower cover per habitat type over the whole season. Different letters indicate significant differences (*p* < 0.05). (c) Differences in species richness of native and ornamental flowering species per habitat type.

Habitat types showed significant differences in flower richness (glmmTMB: χ^2^ = 1250.81; df = 4, *p* < 0.001) and flower cover (glmmTMB: χ^2^ = 200.1; df = 4, *p* < 0.001) with highest flower richness and cover in cemeteries, lowest in fallows and green areas, and intermediate values in house and farmhouse gardens (Figure 2a,b and Appendix S1: Table S1). Variation within habitat types was largest for house and farmhouse gardens, with the best gardens providing more than 200 flowering plant species, while the flower‐poorest garden provided only 25 flowering plant species. Moreover, we found an interaction between habitat type and floral status (native vs. ornamental; cover: χ^2^ = 204.79; df = 4, *p* < 0.001; richness: χ^2^ = 433.65; df = 4, *p* < 0.001) indicating that ornamental plants made up 10% in fallows and green areas, 52% in cemeteries, and 35% and 32% in farmhouse and house gardens, respectively (Figure 2c, Appendix S1: Table S1).

### Landscape effects on pollinator abundance and richness

**FIGURE 3:**
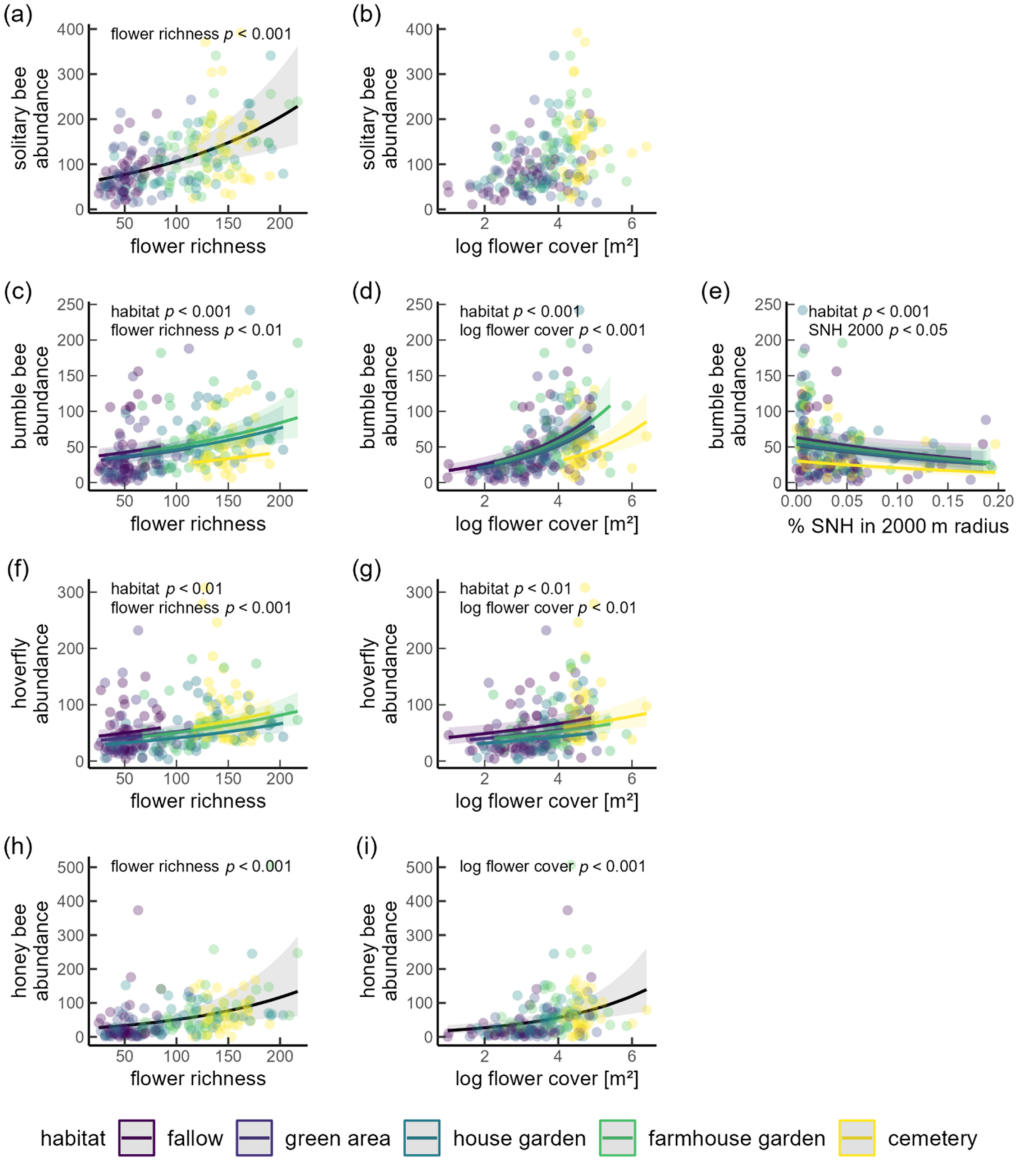
Effects of habitat type, flower richness, flower cover, and percentage of semi‐natural habitats (SNH) on the species abundance of (a, b) solitary bees, (c–e) bumble bees, (f, g) hoverflies, and (h, i) honey bees. Flower cover is log‐transformed. Only significant SNH effects are presented. Shadows indicate the 95% confidence intervals. Prediction lines are not shown if fixed variables had no significant effects.

**FIGURE 4:**
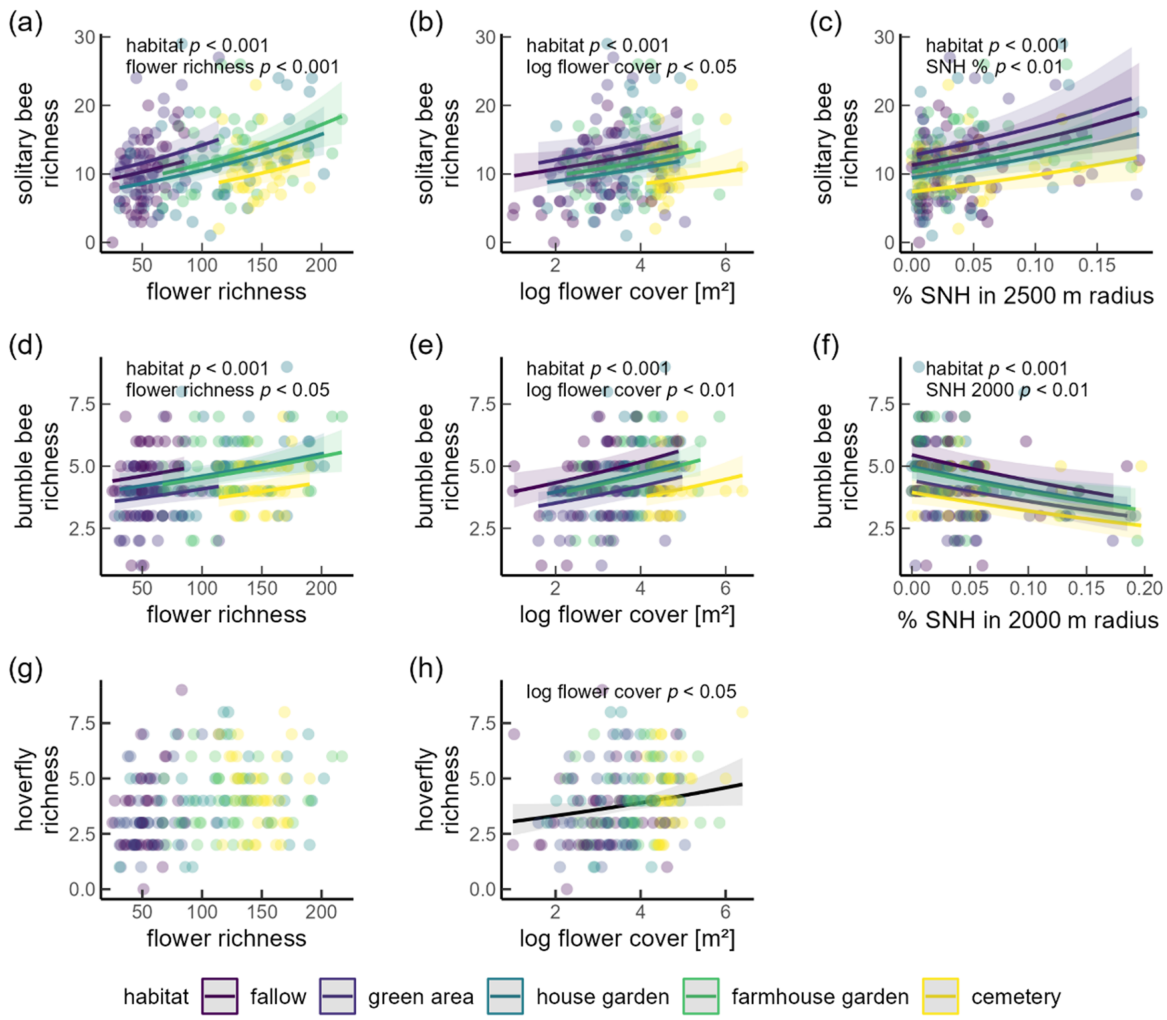
Effects of habitat type, flower richness, flower cover, and percentage of semi‐natural habitats (SNH) on the species richness of (a–c) solitary bees, (d–f) bumble bees, and (g, h) hoverflies. Flower cover is log‐transformed. Only significant SNH effects are presented. Shadows indicate the 95% confidence intervals. Prediction lines are not shown if fixed variables had no significant effects.

For all groups, AIC comparisons indicated consistent effects of SNH and flower cover across habitats. The proportion of semi‐natural habitats was positively related to solitary bee richness, and negatively to bumble bee abundance and richness (Figure 3 and 4). The proportion of semi‐natural habitat had the largest explanatory power on larger scales (solitary bee richness >1500 m, bumble bee abundance >1500 m, bumble bee richness 1000–2500 m) (Appendix S1: Section S3). With an increase of SNH from 1% to 15%, the richness of solitary bees increased by 49% (Figure 4, Table 1). The proportion of semi‐natural habitats had no effect on honey bees or hoverflies and solitary bee abundance, across all considered scales (Figure 3, Table 1). Likelihood ratio tests indicated that habitat‐dependent effects on solitary bee richness and abundance were not supported at smaller scales; only at the largest scales (>2500 m), marginal responses of species richness were detected, with SNH effects being slightly stronger in house and farmhouse gardens than in other habitats (Appendix S1: Sections S3 and S4). These discrepancies between AIC‐ and LRT‐based inference likely reflect small but detectable improvements in model fit that were not sufficient to outweigh the AIC penalty for increased model complexity.

**TABLE 1:** ANOVA summary output of generalized linear mixed‐effects models explaining the effects of habitat type, flower richness, flower cover, mean annual temperature, and percentage of semi‐natural habitat (SNH) in the village surrounding on pollinator abundance and richness for wild pollinators. For SNH, the radius given in parentheses indicates the spatial scale (m) that resulted in the best‐fitting model and was therefore selected for inclusion in the final analysis.

Response	Predictors	Χ^2,^ ^a^	df^b^	*p* value	*R* ^2^ (marg)^c^	*R* ^2^ (cond)^d^
Solitary bee abundance	habitat	2.39	4	0.664	0.33	0.43
	**flower richness**	**21.70**	**1**	<0.001***		
	**temperature**	**5.74**	**1**	<0.05*		
	log(flower cover)	2.26	1	0.133		
	SNH (750m)	0.39	1	0.531		
Bumble bee abundance	**habitat**	**28.95**	**4**	<0.001***	0.46	0.60
	**flower richness**	**8.95**	**1**	<0.01**		
	temperature	0.42	1	0.519		
	**log(flower cover)**	**43.10**	**1**	<0.001***		
	**SNH (2000m)**	**5.23**	**1**	<0.05*		
Hoverfly abundance	**habitat**	**15.41**	**4**	<0.01**	0.34	0.65
	**flower richness**	**11.34**	**1**	<0.001***		
	**temperature**	**7.54**	**1**	<0.01**		
	**log(flower cover)**	**8.43**	**1**	<0.01**		
	SNH (1500m)	0.80	1	0.372		
Honey bee abundance	habitat	8.77	4	0.067	0.40	0.52
	**flower richness**	**12.00**	**1**	<0.001***		
	temperature	0.14	1	0.708		
	**log(flower cover)**	**14.00**	**1**	<0.001***		
	SNH (2500m)	0.92	1	0.337		
Solitary bee richness	**habitat**	**18.81**	**4**	<0.001***	0.29	0.32
	**flower richness**	**12.87**	**1**	<0.001***		
	temperature	0.15	1	0.695		
	**log(flower cover)**	**4.59**	**1**	<0.05*		
	**SNH (2500m)**	**8.61**	**1**	<0.01**		
Bumble bee richness	**habitat**	**29.10**	**4**	<0.001***	0.10	0.13
	**flower richness**	**6.55**	**1**	<0.05*		
	temperature	0.01	1	0.919		
	**log(flower cover)**	**10.21**	**1**	<0.01**		
	**SNH (2000m)**	**9.16**	**1**	<0.01**		
Hoverfly richness	habitat	2.59	4	0.628	0.06	0.09
	flower richness	1.52	1	0.217		
	**temperature**	**12.29**	**1**	<0.001***		
	**log(flower cover)**	**3.91**	**1**	<0.05*		
	SNH (250m)	2.77	1	0.096		

*Note*: Boldface indicates statistically significant predictors. Asterisks indicate significance level: *p < 0.05, **p < 0.01, ***p < 0.001.

^a^ Chi‐square.

^b^ Degrees of freedom.

^c^ *R* ^2^ marginal.

^d^ *R* ^2^ conditional.

### Effects of habitat type and floral resources on pollinator abundance and richness

For most pollinator groups and habitat types, pollinator richness and abundance increased with both increasing flower richness and flower cover. Overall, increases in flower cover consistently led to higher pollinator abundance and richness across all habitat types. There was no evidence of a habitat × flower cover interaction for bumble bees, hoverflies, or honeybees. For solitary bees, a very weak interaction was supported only in models including %SNH at large spatial scales (>2500 m), however, models without interaction and including %SNH at smaller spatial scales were equally supported, indicating that the model is not improved at larger scales and the interaction effect can be considered negligible (Appendix S1: Sections S3 and S4). Nonetheless, habitat type had an additional additive effect in almost all pollinator groups with the exception of honey bees for which habitat had only marginal effects (Table 1; Appendix S1: Table S10). Additive effects of habitat type, flower richness, and flower cover were found on both bumble bee richness and abundance, solitary bee richness, and hoverfly abundance (Figures 3 and 4): Solitary bee richness in green areas and fallows, and bumble bee richness and abundance in fallows were higher than expected from the relatively low flower richness in these habitats (Figure 2, 3, 4; Appendix S1: Table S10, Figure S3). This was evident in the higher or equally high pollinator abundance and richness compared to other habitats with higher flower richness, such as house gardens, farmhouse gardens, and particularly cemeteries. In contrast, cemeteries showed relatively low pollinator richness and abundance for most groups despite high flower richness and cover in this habitat type (Figures 2 and 3; Appendix S1: Table S3).

Wild pollinator groups exhibited a general negative trend in response to increasing proportions of ornamental cover, while honey bees exhibited a positive response. Richness of solitary bee species, but not of the other wild pollinator taxa, significantly declined with higher proportion of ornamental cover (Appendix S1: Section S6).

### Attractiveness of different plant genera for pollinators

A total of 583 plant genera were included in this analysis, of which 374 genera were visited by 38,620 pollinators over the whole study period. Solitary bees visited 71 genera, bumble bees 76, hoverflies 67, and honey bees 63 genera more often than expected based on observed flower cover. Even more genera were less frequently visited than expected. 89 plant genera belonged to these non‐preferential genera for solitary bees, 139 for bumble bees, 122 for hoverflies, and 136 for honey bees. In total, 254 genera were visited by all pollinator groups as frequently as expected, that is, visitation frequency was consistent with the null model (Maihoff & Schulze, 2025).

**FIGURE 5:**
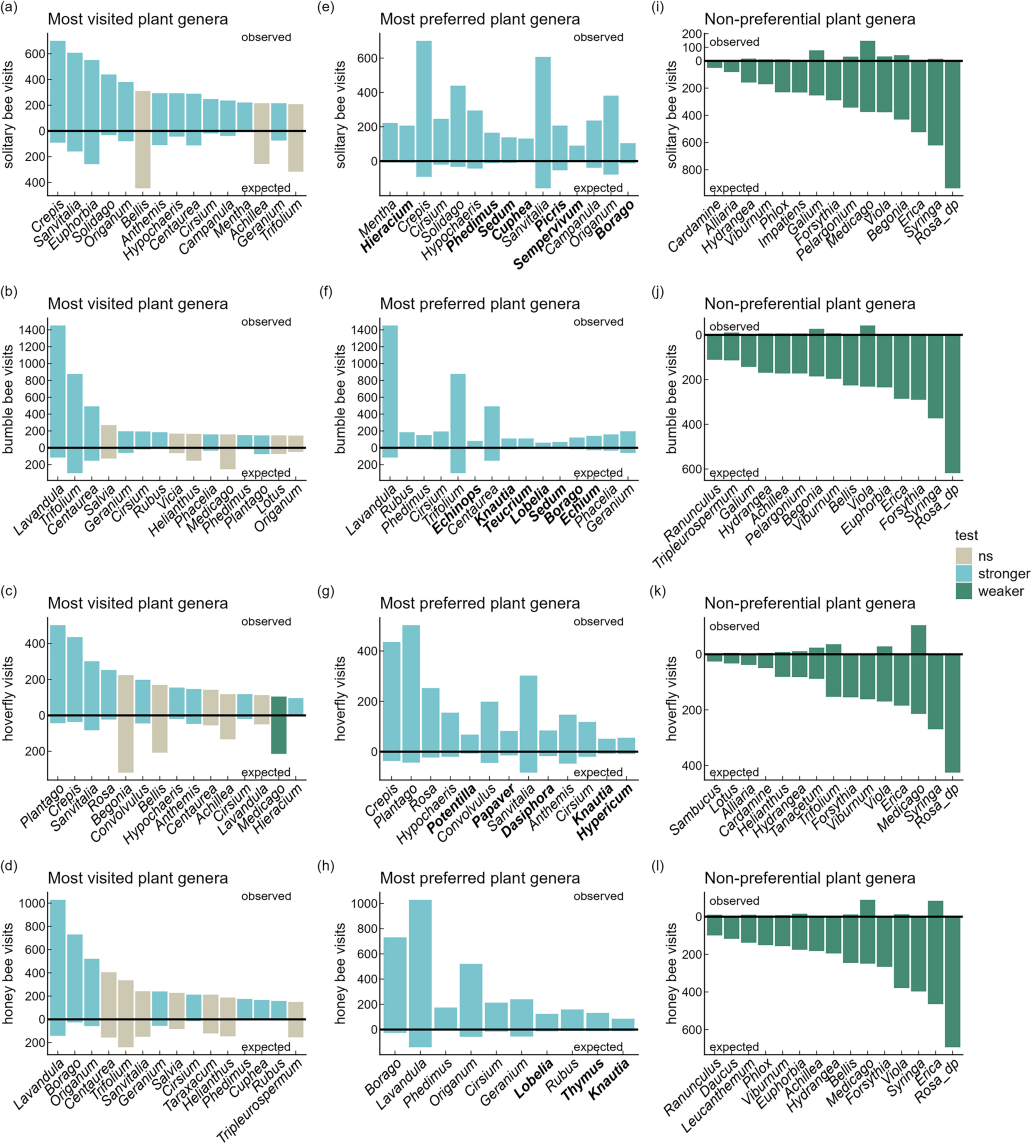
List of the 15 most visited, most preferred, and non‐preferential plant genera for the pollinator groups’ solitary bees, bumble bees, hoverflies, and honey bees. The most visited plant genera are the summed observed visits for the whole study period, the most preferred plant genera are based on the highest standardized effect size (SES) calculated by the null model, and the non‐preferential plant genera are based on the lowest SES calculated by the null model. To prevent a disproportionally high display of single visitation events on small flowers, a threshold of at least 5 observed visits for the most visited plant genera was applied. The most preferred genera in bold are not among the most visited genera.

The most visited plant genera were *Crepis* and *Sanvitalia* for solitary bees, *Lavandula* and *Trifolium* for bumble bees, *Plantago* and *Crepis* for hoverflies, and *Lavandula* and *Borago* for honey bees (Figure 5a,d,g,j). Interestingly, some genera among the top‐15 most visited plant species were not more frequently visited than expected and got a high number of visits only because of their relatively high flower cover (e.g., solitary bees' visits on *Bellis* and *Achillea*, bumble bees' on *Helianthus* and *Medicago*, hoverflies' on *Begonia*, honey bees' on *Trifolium*).

In a second step, we identified the top‐15 most preferred plant genera for each pollinator group, that is, those genera where the observations exceeded expectations most based on their flower cover (Figure 5b,e,h,k). Here, we found three to seven highly preferred genera per pollinator group that were not among the top‐15 most visited genera. These genera had relatively low flower covers (and thus only few expected visits), but were nevertheless very attractive (e.g., *Borago*, preferred by solitary bees, bumble bees, and honey bees).

Bumble bees and honey bees had great overlap in their preferences, sharing ten and eight genera of the most visited and most preferred plant genera, respectively. Similarly, solitary bees and hoverflies shared eight and four genera, while solitary bees and bumble bees shared five and four genera of the most visited and most preferred plant genera. The only genera that were among the top‐15 most visited plants for all pollinator groups were *Centaurea* and *Cirsium*. Only one genus, *Cirsium*, was shared among the top‐15 most preferred genera for all pollinator groups.

Contrasting the most preferred plant genera, we also identified the top‐15 non‐preferential plant genera, that is, those genera where the observations fell most below expectations based on their flower cover (Figure 5c,f,i,l). Seven genera were shared for all pollinator groups, whereas one to five genera were linked to specific pollinator groups. For all pollinator groups, double‐petaled *Rosa*, *Syringa*, and *Erica* were among the least‐preferred top‐15 non‐preferential plant genera.

## DISCUSSION

Our study highlights that villages can support unexpectedly high pollinator diversity and abundance, even for conservation‐priority groups such as pollinators. We found a total richness of 208 wild bee species, including bumble bees, representing 40% of Bavaria's known species with 22.5% classified as endangered (Voith et al., 2021). Comparatively lower was hoverfly richness with 56 species which make up 14% of the Bavarian species (Ssymank et al., 2011). Importantly, habitat types within villages differed in pollinator richness and abundance, and, in the case of solitary bees, benefited from higher proportion of semi‐natural habitat in the surrounding landscape. Local differences among habitat types were partly explained by differences in flower richness and abundance. Additive effects of habitat type and flower resources strongly suggest that other habitat differences, for example, the attractiveness of plant communities and presumably the availability of nesting sites additionally contributed to differences in pollinator richness and abundance among habitat types. Our study highlights the underexploited potential of village habitats for pollinator conservation and provides important hands‐on information for the targeted management of public and private areas.

### Landscape composition modulates pollinator communities in village habitats

Habitats within villages are interconnected with the surrounding landscape environment. The positive effect of semi‐natural habitats on solitary bee richness suggests that these habitats contribute to the species pool of the villages, for example, by serving as source habitats with high‐quality nesting structures for solitary wild bees (Maurer et al., 2022; Papanikolaou et al., 2017). Given the relatively short foraging distances of most solitary bees (<1000 m), regular foraging flights are unlikely to drive this effect (Gathmann & Tscharntke, 2002, Zurbuchen et al., 2010). Interestingly, the negative effects detected for bumble bees suggest that, when foraging ranges permit exploration of resources and nesting sites over larger distance (Hemberger & Williams, 2025), these high‐quality habitats may be preferred over village habitats. In contrast, in landscapes with low proportion of semi‐natural habitats, villages may become important refuge habitats for bumble bees. Because the habitat‐dependent effects we observed were limited to wild bees at the largest scales and were small, conservation planning can prioritize maintaining and enhancing semi‐natural habitats at the landscape level rather than focusing narrowly on the surrounding of specific village habitat types.

### Effects of habitat types and floral resources

We found an extremely high number of 1258 different plant species in our study. Habitat types differed in flower availability with cemeteries displaying the highest flower richness and flower cover, whereas fallows and green areas showed the lowest flower richness and flower cover. Aesthetic preferences, economic conditions, and the number of plant species currently available in garden markets influence human decision‐making regarding floral composition within urban habitats (Aronson et al., 2016) and in this case also in rural settlements like villages. These decisions lead increasingly to the introduction of ornamental plant species (Reichard & White, 2001), and both plant selection and the active management of designed plantings—aimed at facilitating the coexistence of a wide variety of flowering species—play a crucial role in shaping species‐rich habitats. This intentional diversification can explain the generally higher richness of plant species in urban areas and villages than in agricultural areas (Udy et al., 2020; Wania et al., 2006). As we could show, this is especially true in habitats such as gardens and cemeteries, where personal preferences about the types of plants used in beds and plantings play an important role. On the other hand, fallows, with little human influence, tended to have a spontaneous, less species‐rich vegetation compared to other village habitats. Similarly, we found a relatively low plant richness in green areas, where the social convention is that everything must be “tidy” with often‐mowed short lawns. In house gardens, the range of plant species, native and ornamental, was particularly wide across the 40 gardens surveyed. This shows that house gardens can be extremely species‐rich and, on the other hand, it indicates that there are still many house gardens where this potential is far from being realized. The same, although slightly less pronounced, is true of farmhouse gardens.

In line with other studies, we demonstrate positive effects of floral resource richness and abundance on the richness and abundance of all pollinator groups (Scheper et al., 2013; Steffan‐Dewenter & Tscharntke, 2001). This suggests that pollinators benefited more strongly from a combination of high flower richness and high flower cover than from high flower richness or high flower cover alone. This effect is particularly evident in farmhouse and house gardens. Both showed high variability in flower richness, cover, and species identity. These gardens ranged from insect‐friendly management—characterized by less mowing and weeding, allowing for greater native flowering plant diversity—to a primarily aesthetic approach, featuring a higher proportion of highly bred ornamental flowers and a carefully maintained, regularly mown lawn without flowers. This variability in garden management resulted in a corresponding variation in pollinator abundance and richness, mirroring the differences in floral richness and availability.

Furthermore, we found additive effects of habitat type and flower resources on both or either abundance and richness of all pollinator groups, except for honey bees. These additional effects of habitat type suggest that, even if pollinators increased with increasing flower resources, some pollinator groups responded in unexpected ways. Based on the low flower availability in fallows and green areas and the high flower availability in cemeteries, we expected correspondingly low or high pollinator abundance and richness. However, this was not the case for hoverflies, solitary bees, and bumble bees, as fallows and green areas exhibited either a higher‐than‐expected pollinator abundance, richness, or both, while cemeteries showed either a lower‐than‐expected abundance, richness, or both. Green areas and, in particular, fallows were usually not planted and were dominated by spontaneous wild native plant species, which are positively associated with pollinator visits (Fukase, 2016). Moreover, the higher‐than‐expected richness in fallows and green areas suggests that variables other than flower richness and flower cover, such as nesting resources, might have played a role (Fortel et al., 2016; Harmon‐Threatt, 2020; Potts et al., 2005). Most of the bee species nest in the ground and benefit from bare ground and, in the case of bumble bees, from abandoned rodent burrows. Both features may be more abundant in fallows and green areas than in gardens and cemeteries. In addition, soil tillage is less common in fallows and green areas than in gardens and cemeteries, as well as the use of bark mulch to cover bare soil in plant beds.

Our results suggest that nesting resources made fallows and green areas more valuable habitats despite their low supply of flower resources. In contrast, cemeteries showed lower pollinator abundance and richness than expected from the high flower richness and cover in this habitat type. This discrepancy may be linked to planting choices influenced by religious and traditional aesthetic preferences (Sallay et al., 2023), resulting in a higher share of ornamental plants, often assumed to be less beneficial for pollinators (Fukase, 2016). While we observed a clear negative effect of the proportion of ornamental cover on solitary bee richness and a generally negative but non‐significant trend on other wild pollinators across all habitats (Appendix S1: Section S6), indicating at maximum a weak preference for native plants, the proportion of ornamental plants in cemeteries only partly explains why higher flower availability did not consistently translate into increased pollinator abundance and richness. Surprisingly, especially bumble bees, generalist species known to forage on complex flower shapes (Sponsler et al., 2022) and to cope relatively well with non‐native plants (Martini et al., 2025, see also Appendix S1: Section S6), showed a low abundance and richness in cemeteries. Although limited nesting sites may play a role, we propose that plant cultivation is an influential factor here. Cultivated plants, whether native or non‐native, often provide less nectar and pollen (Smitley et al., 2024; Strzałkowska‐Abramek, 2019), and in strong aesthetic‐driven plantings, as in cemeteries, the prevalence of low‐reward, pollinator‐unattractive plants likely increases (Garbuzov et al., 2017; Garbuzov & Ratnieks, 2014). For bumble bees, with a high metabolic cost of flight (Goulson, 2010), foraging in low‐reward areas may be too energetically expensive. Nevertheless, if ornamental plants are not highly cultivated, pollinators can also benefit from non‐native flowering plant species and the extension of the flowering season that fills potential resource gaps (Matteson & Langellotto, 2011; Salisbury et al., 2015), which in our study may explain the lack of a strong negative effect of a high proportion of ornamental plants on pollinator abundances across all habitats. Still, as the example of cemeteries has shown, it seems necessary to allow spontaneous vegetation of wild plants and include non‐cultivated plants in the plantings and not rely exclusively on ornamental and cultivated species to promote high abundance and richness of pollinators.

Hoverfly richness was not affected by habitat type, nor flower richness. This can be explained by larval requirements. For example, predatory hoverfly larvae require species‐specific insect prey (Dziock, 2013; Rocha et al., 2018), whereas saproxylic hoverflies require trunk cavities or rot holes (Maritano et al., 2024; Meyer et al., 2009; Reemer, 2005). This suggests that the availability of prey, suitable dead wood, and large trees—rather than habitat type or flower richness—may be the key determinant of species richness. In contrast to wild bees, honey bees were largely unaffected by habitat type, but increased with flower availability only. This finding supports the idea that the additive effects of habitat type observed in wild bees may result from differences in the availability of nesting sites. In the case of honey bees, abundance might rather depend on the location of apiaries than on habitat type (Steffan‐Dewenter & Kuhn, 2003).

### Pollinator preferences

Based on 38,620 pollinator–plant interactions, we identified the most visited, most preferred (corrected for plant abundance), and non‐preferential plant genera for four major pollinator groups. With our results, we can provide village residents and stakeholders differentiated information to improve the value of village habitats for pollinators. Our results are in line with the three most frequently mentioned plant genera attractive to pollinators in the lists evaluated by Garbuzov and Ratnieks (2014), *Origanum*, *Sedum*, and *Solidago*, but only for solitary bees. The most visited and most preferred plant genus for bumble bees, *Lavandula* is still mentioned in 13 of 15 lists, whereas the second most visited genus for bumble bees, *Trifolium*, is not mentioned at all. However, the recently published paper from Kuppler et al. (2023) gives a detailed description of the favorite plant species for wild bees in Germany and their similar approach revealed many overlaps with our findings, for example, the preference of wild bees for *Hieracium*, *Picris*, *Hypochaeris*, and *Origanum*. This overlap may highlight the strong affinity of oligolectic bees for Asteraceae (Zurbuchen & Müller, 2012) and their preference for yellow color, as observed in bees of the genus *Lasioglossum* (Heuel et al., 2024). While Kuppler et al. (2023) focused on native plant species in flower strips in agricultural landscapes, our study uncovered non‐native surprise winners in village habitats such as *Cuphea* and *Sanvitalia* as the most preferred ornamental plant genera for solitary bees. To our knowledge, both genera have not been mentioned in the literature as pollinator‐friendly yet. Moreover, different pollinator species also display different preferences, depending on species traits, such as proboscis size (Moquet et al., 2018; Stang et al., 2009), and therefore prefer different floral morphotypes (Sponsler et al., 2022). Groups with similar traits, such as honey bees and bumble bees, which are both polylectic, central‐place foragers, and have rather similar proboscis lengths, share many preferred genera, as shown in grasslands (Cappellari et al., 2022). In contrast, groups with different traits, such as bumble bees and hoverflies, which differ in proboscis length (Fontaine et al., 2005), do not share as many frequently visited or preferred genera. For example, the deep corolla of *Lavandula* is most effectively utilized by medium‐ and long‐tongued species, as evidenced by the strong preference for this genus among honey bees and bumble bees (Balfour et al., 2013). Differences between pollinator groups with similar traits can arise due to competitive constraints, as seen in the preference of honey bees—but not bumble bees—for *Borago*. While both species visit *Lavandula*, honey bees tend to shift to *Borago* when competition intensifies, likely because bumble bees forage even more efficiently on *Lavandula* (Balfour et al., 2013). Hoverflies preferred *Plantago*, which underlines that this pollinator group often uses pollen from wind‐pollinated plant species (Bastian, 1986). Even if plant lists recommended for pollinators are easily available, they often lack information on how the data were collected or attractiveness measured (Garbuzov & Ratnieks, 2014). In addition, most focus only on a single group or overlook the important differentiation between groups, which is essential knowledge to meet their specific needs.

Despite the importance of top genera found, it is necessary to keep the characteristics and origins of the plants in mind when choosing suitable pollinator plants. Some *Cirsium* species, for example, are well known as notorious weeds for gardens and agricultural lands (Glinwood et al., 2004) and therefore need to be handled with care. The same applies to non‐native invasive plants, like *Solidago canadensis* (Zhang et al., 2009), which are nevertheless gladly welcomed nectar and pollen resources for solitary bees.

## CONCLUDING IMPLICATIONS FOR MANAGEMENT

The large numbers of pollinator species and their high abundance highlight the importance of villages as a vital habitat for wild pollinators. Particularly within landscapes affected by land‐use change and agricultural intensification, they offer unexploited conservation opportunities amid an alarming decline in biodiversity (Wagner et al., 2021). Moreover, our results highlight that village habitats are ecologically interconnected with the surrounding landscape: semi‐natural habitats act as important source areas for solitary bees, while bumble bees may rely more strongly on villages in landscapes where semi‐natural habitats are scarce. However, increasing numbers of intensively managed gardens (specifically house gardens) with frequently mown lawns and gravel gardens as well as intensively mown green areas underpin that villages do not yet live up to their full potential. Our study highlights in which village habitats which management measures can be used in a particularly promising way to promote pollinators. For example, in fallows and green areas, even small increases in the supply of native flowers lead to large effects, whereas in cemeteries, which are rich in flowering ornamental species, it is crucial to select more plants that actually provide nectar and pollen resources for pollinators. Farmhouse gardens, and especially house gardens, show a wide range from highly diverse to very poor in diversity, serving as examples of what is achievable in a garden. Even in small spaces, a dedicated citizen can host a wide variety of pollinators with appropriate ecological care, pollinator‐friendly plants, and suitable nesting opportunities. On the other hand, a large garden can become a veritable desert if it is not properly maintained, or if it is ecologically inappropriate due to restrictive social conventions. We recommend that gardens with a low pollinator richness add pollinator‐friendly plantings from our list where possible or maintain existing ones. Hereby, our list shows that carefully chosen ornamental plants with high aesthetic value and high diversity are not necessarily contradictions. Above all, avoid the fear of “messing up” the villages: Instead of constantly weeding and mowing, allow spontaneous vegetation with native plants to grow and create more nesting sites by leaving areas of bare ground rather than reseeding the lawn. Our list of most visited, most preferred, and non‐preferential plant genera will help garden owners and local stakeholders to select the appropriate plants to effectively conserve and promote different pollinator groups. With our recommendations, we offer villages the opportunity to fill habitat gaps in the agricultural environment, thereby realizing their currently untapped potential to conserve a significant proportion of the regional pollinator fauna. Yet, this potential can only be fully realized if village habitat enhancement goes hand in hand with safeguarding and restoring semi‐natural habitats.

## AUTHOR CONTRIBUTIONS

Andrea Holzschuh and Ingolf Steffan‐Dewenter conceived the ideas and designed the study. Sonja Schulze, Daniela Kessner‐Beierlein, Alicia Bender, and Annika Schöninger collected the field data. Sonja Schulze and Fabienne Maihoff led statistical analysis and the writing of the first draft of the manuscript. Jie Zhang harmonized the GIS data and calculated the landscape metrices. All authors contributed critically to the drafts and gave their final approval for publication.

## CONFLICT OF INTEREST STATEMENT

The authors declare no conflicts of interest.

## Supporting information


Appendix S1.

## Data Availability

Data and code (Maihoff & Schulze, 2025) are available in Figshare at https://doi.org/10.6084/m9.figshare.28668011.v1.
