# Supplementary material for: More than flowers: Habitat type, floral resources, and landscape context shape pollinator communities in villages
Source: Ecol Appl. 2026 Feb 24;36(1):e70190. doi: 10.1002/eap.70190 (PMC12931365; doi:10.1002/eap.70190)
Supplement: Supplementary file 1 — Appendix S1. [file EAP-36-e70190-s001.pdf]

Appendix S1

More than flowers: Habitat type, floral resources, and landscape context shape pollinator communities in villages

Sonja Schulze, Fabienne Maihoff, Jie Zhang, Daniela Kessner-Beierlein, Alicia Bender, Annika Schöninger, Andrea Holzschuh, Ingolf Steffan-Dewenter

Ecological Applications

Section S1: Habitat types

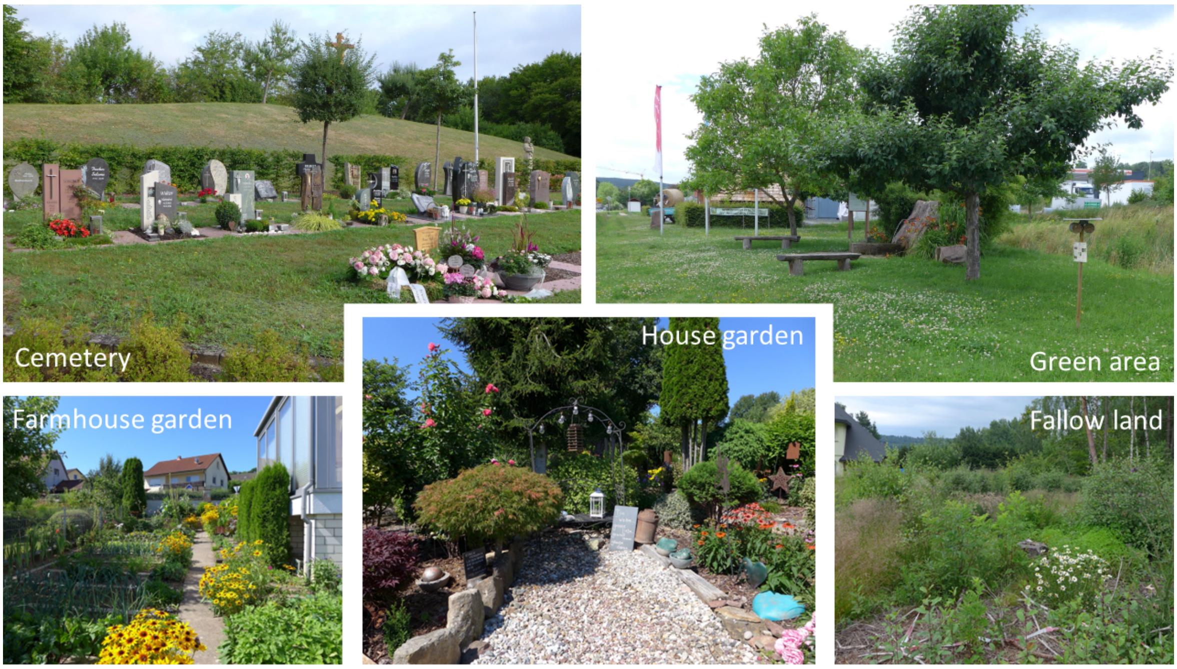

Figure S1. Examples of the five habitat types used in this study. *Photo credits: Sonja Schulze.*

Section S2: Effects of habitat type on flower richness, cover and floral status

Table S1. Post-hoc comparisons of habitat types on floral composition*: The table presents estimated marginal means (Estimated mean) for different habitat types and their corresponding standard errors (SE), confidence intervals (LCL and UCL), and statistical groupings based on Tukey's HSD test per factor. Habitat types with the same grouping symbol (e.g., "a", "b", "c") were not significantly different from each other (p > 0.05). Results for flower, ornamental and native cover and for native and ornamental richness, are given on the log (not the response) scale.*

Response	Habitat	Estimated mean	SE	DF	LCL	UCL	Letters
	fallow	3.10	0.15	194	2.80	3.40	a
	green area	3.13	0.12	194	2.89	3.36	a
flower cover	house garden	3.67	0.12	194	3.43	3.90	b
	farmhouse garden	3.84	0.13	194	3.58	4.11	b
	cemetery	4.66	0.07	194	4.53	4.80	c
	fallow	52.75	2.20	194	48.41	57.09	a
	green area	55.45	3.21	194	49.12	61.78	a
flower richness	house garden	117.43	6.69	194	104.23	130.62	b
	farmhouse garden	124.33	6.35	194	111.81	136.84	b
	cemetery	147.43	3.02	194	141.47	153.38	c
	fallow	3.28	0.16	Inf	2.96	3.59	a
	green area	3.11	0.16	Inf	2.79	3.43	a
native cover	house garden	3.08	0.16	Inf	2.76	3.40	a
	farmhouse garden	3.32	0.16	Inf	3.00	3.64	a
	cemetery	3.35	0.17	Inf	3.02	3.67	a
	fallow	-0.25	0.27	Inf	-0.78	0.29	a
	green area	0.72	0.24	Inf	0.25	1.19	b
ornamental cover	house garden	2.49	0.18	Inf	2.14	2.84	c
	farmhouse garden	3.00	0.17	Inf	2.66	3.34	c
	cemetery	4.16	0.14	Inf	3.88	4.44	d
	fallow	3.77	0.06	Inf	3.66	3.87	a
	green area	3.75	0.06	Inf	3.64	3.86	a
native richness	house garden	4.15	0.05	Inf	4.06	4.25	b
	farmhouse garden	4.19	0.05	Inf	4.09	4.28	b
	cemetery	4.05	0.05	Inf	3.95	4.15	b
	fallow	0.78	0.19	Inf	0.41	1.15	a
	green area	1.26	0.16	Inf	0.95	1.57	a
ornamental richness	house garden	3.43	0.06	Inf	3.30	3.55	b
	farmhouse garden	3.57	0.06	Inf	3.45	3.68	b
	cemetery	4.11	0.05	Inf	4.01	4.21	c

Section S3: Model selection for pollinator abundance and richness at multiple spatial scales of semi-natural-habitat (SNH)

This section presents the results of generalized linear mixed-effects model (GLMM) comparisons for pollinator abundance and species richness in relation to seminatural habitat (SNH) cover across multiple spatial scales, as well as habitat-dependent effects of flower cover and SNH proportion. For each response variable, we report AICc values across scales, ΔAICc within each scale, marginal and conditional R^2, and likelihood ratio test (LRT) p-values for baseline and interaction models, highlighting the best-supported models and any scale- or habitat-dependent effects. Overall no habitat-dependent effects were supported. For wild bees, there was only weak indication of a habitat-dependent interaction, and this was limited to models that included semi-natural habitat at large spatial scales (> 2500m) (see also Section S6).

Table S2. Model comparison results for solitary bee abundance *in relation to semi-natural habitat (SNH) cover at multiple spatial scales (250–3000 m). Candidate models included: baseline model without interactions (non), habitat × floral cover (int1), habitat × SNH (int2), and the full interaction model including both terms (both). Shown are small-sample corrected Akaike Information Criterion (AICc), differences from the best model (ΔAICc), marginal and conditional R^2 values, and p-values from likelihood ratio tests (LRT) comparing each interaction model to the baseline. The best-supported model per scale (smallest AICc) are in bold. Rows shaded in grey indicate the best supported model overall scales (AICc < 2 or significant LRT).*

Response	SNH scale in m	Model	AICc	delta_AICc	R2 marg/cond	LRT_pval
**solitary bee abundance**	**250**	**non**	2,143.87	0.00	0.33/0.43	
solitary bee abundance	250	int1	2,144.76	0.88	0.36/0.46	0.08
solitary bee abundance	250	int2	2,150.80	6.93	0.34/0.43	0.69
solitary bee abundance	250	both	2,152.18	8.31	0.37/0.46	0.23
**solitary bee abundance**	**500**	**non**	2,143.84	0.00	0.33/0.43	
solitary bee abundance	500	int1	2,144.74	0.90	0.36/0.46	0.08
solitary bee abundance	500	int2	2,150.40	6.56	0.34/0.44	0.62
solitary bee abundance	500	both	2,151.66	7.82	0.37/0.48	0.20
**solitary bee abundance**	**750**	**non**	2,143.48	0.00	0.33/0.43	
solitary bee abundance	750	int1	2,144.48	1.00	0.36/0.46	0.08
solitary bee abundance	750	int2	2,148.26	4.77	0.35/0.45	0.35
solitary bee abundance	750	both	2,149.00	5.51	0.38/0.48	0.10
**solitary bee abundance**	**1000**	**non**	2,143.51	0.00	0.33/0.43	
solitary bee abundance	1000	int1	2,144.43	0.92	0.36/0.46	0.08
solitary bee abundance	1000	int2	2,147.95	4.44	0.35/0.45	0.31
solitary bee abundance	1000	both	2,148.62	5.11	0.38/0.49	0.09
**solitary bee abundance**	**1500**	**non**	2,143.78	0.00	0.33/0.43	
solitary bee abundance	1500	int1	2,144.66	0.88	0.36/0.46	0.08
solitary bee abundance	1500	int2	2,147.97	4.19	0.35/0.45	0.29
solitary bee abundance	1500	both	2,148.72	4.94	0.38/0.49	0.09
**solitary bee abundance**	**2000**	**non**	2,143.81	0.00	0.33/0.43	
solitary bee abundance	2000	int1	2,144.65	0.84	0.36/0.46	0.08
solitary bee abundance	2000	int2	2,147.62	3.81	0.35/0.45	0.25
solitary bee abundance	2000	both	2,148.20	4.39	0.38/0.49	0.07
**solitary bee abundance**	**2500**	**non**	2,143.63	0.00	0.33/0.43	
solitary bee abundance	2500	int1	2,144.42	0.79	0.37/0.46	0.08
solitary bee abundance	2500	int2	2,146.47	2.84	0.35/0.46	0.17
solitary bee abundance	2500	both	2,146.54	2.91	0.39/0.49	0.04
**solitary bee abundance**	**3000**	**non**	2,143.54	0.00	0.33/0.43	
solitary bee abundance	3000	int1	2,144.38	0.83	0.37/0.46	0.08
solitary bee abundance	3000	int2	2,145.66	2.11	0.35/0.46	0.13
solitary bee abundance	3000	both	2,145.42	1.88	0.39/0.49	0.03

***Table S3. Model comparison results for bumble bee abundance** in relation to semi-natural habitat (SNH) cover at multiple spatial scales (250–3000 m). Candidate models included: baseline model without interactions (non), habitat × floral cover (int1), habitat × SNH (int2), and the full interaction model including both terms (both). Shown are small-sample corrected Akaike Information Criterion (AICc), differences from the best model (ΔAICc), marginal and conditional R^2 values, and p-values from likelihood ratio tests (LRT) comparing each interaction model to the baseline. The best-supported model per scale (smallest AICc) are in bold. Rows shaded in grey indicate the best supported model overall scales (AICc < 2 or significant LRT <0.05).*

Response	Predictor	Model	AICc	delta_AICc	R2 marg/cond	LRT_pval
**bumble bee abundance**	**250**	**non**	1,865.19	0.00	0.44/0.58	
bumble bee abundance	250	int1	1,866.13	0.93	0.47/0.60	0.08
bumble bee abundance	250	int2	1,872.45	7.25	0.44/0.59	0.74
bumble bee abundance	250	both	1,873.80	8.61	0.47/0.61	0.25
**bumble bee abundance**	**500**	**non**	1,865.44	0.00	0.44/0.58	
bumble bee abundance	500	int1	1,866.30	0.86	0.47/0.60	0.08
bumble bee abundance	500	int2	1,872.93	7.49	0.44/0.59	0.79
bumble bee abundance	500	both	1,874.42	8.99	0.47/0.60	0.28
**bumble bee abundance**	**750**	**non**	1,864.91	0.00	0.44/0.58	
bumble bee abundance	750	int1	1,865.66	0.75	0.47/0.60	0.08
bumble bee abundance	750	int2	1,871.93	7.02	0.45/0.59	0.70
bumble bee abundance	750	both	1,872.86	7.95	0.48/0.61	0.21
**bumble bee abundance**	**1000**	**non**	1,864.60	0.00	0.44/0.58	
bumble bee abundance	1000	int1	1,865.46	0.85	0.47/0.60	0.08
bumble bee abundance	1000	int2	1,871.65	7.04	0.45/0.59	0.71
bumble bee abundance	1000	both	1,872.46	7.86	0.48/0.61	0.20
**bumble bee abundance**	**1500**	**non**	1,862.31	0.00	0.45/0.59	
bumble bee abundance	1500	int1	1,862.95	0.64	0.49/0.61	0.07
bumble bee abundance	1500	int2	1,869.26	6.95	0.46/0.60	0.69
bumble bee abundance	1500	both	1,869.18	6.87	0.49/0.61	0.15
**bumble bee abundance**	**2000**	**non**	1,860.39	0.00	0.46/0.60	
bumble bee abundance	2000	int1	1,860.99	0.60	0.49/0.61	0.07
bumble bee abundance	2000	int2	1,867.15	6.76	0.47/0.60	0.65
bumble bee abundance	2000	both	1,866.84	6.45	0.50/0.62	0.14
**bumble bee abundance**	**2500**	**non**	1,861.12	0.00	0.46/0.59	
bumble bee abundance	2500	int1	1,861.58	0.47	0.49/0.61	0.07
bumble bee abundance	2500	int2	1,867.20	6.08	0.46/0.60	0.54
bumble bee abundance	2500	both	1,866.68	5.56	0.50/0.62	0.10
**bumble bee abundance**	**3000**	**non**	1,862.27	0.00	0.45/0.59	
bumble bee abundance	3000	int1	1,862.72	0.45	0.48/0.60	0.07
bumble bee abundance	3000	int2	1,867.95	5.67	0.46/0.60	0.47
bumble bee abundance	3000	both	1,867.37	5.10	0.50/0.61	0.09

***Table S4. Model comparison results for hoverfly abundance** in relation to semi-natural habitat (SNH) cover at multiple spatial scales (250–3000 m). Candidate models included: baseline model without interactions (non), habitat × floral cover (int1), habitat × SNH (int2), and the full interaction model including both terms (both). Shown are small-sample corrected Akaike Information Criterion (AICc), differences from the best model (ΔAICc), marginal and conditional R^2 values, and p-values from likelihood ratio tests (LRT) comparing each interaction model to the baseline. The best-supported model per scale (smallest AICc) are in bold. Rows shaded in grey indicate the best supported model overall scales (AICc < 2 or significant LRT <0.05).*

Response	Predictor	Model	AICc	delta_AICc	R2 marg/cond	LRT_pval
**hoverfly abundance**	**250**	**non**	1,896.55	0.00	0.33/0.65	
hoverfly abundance	250	int2	1,902.64	6.09	0.34/0.66	0.54
hoverfly abundance	250	int1	1,899.57	3.02	0.35/0.66	0.19
hoverfly abundance	250	both	1,906.02	9.47	0.36/0.67	0.31
**hoverfly abundance**	**500**	**non**	1,896.89	0.00	0.33/0.65	
hoverfly abundance	500	int1	1,903.00	6.10	0.34/0.66	0.54
hoverfly abundance	500	int2	1,903.05	6.16	0.34/0.65	0.55
hoverfly abundance	500	both	1,909.31	12.42	0.35/0.66	0.60
**hoverfly abundance**	**750**	**non**	1,896.59	0.00	0.34/0.65	
hoverfly abundance	750	int1	1,902.60	6.01	0.35/0.66	0.53
hoverfly abundance	750	int2	1,902.74	6.15	0.35/0.66	0.55
hoverfly abundance	750	both	1,908.41	11.82	0.35/0.67	0.54
**hoverfly abundance**	**1000**	**non**	1,896.62	0.00	0.34/0.65	
hoverfly abundance	1000	int2	1,902.67	6.06	0.35/0.65	0.53
hoverfly abundance	1000	int1	1,901.89	5.27	0.35/0.66	0.42
hoverfly abundance	1000	both	1,907.43	10.82	0.36/0.67	0.43
**hoverfly abundance**	**1500**	**non**	1,896.12	0.00	0.34/0.65	
hoverfly abundance	1500	int2	1,902.18	6.05	0.35/0.65	0.53
hoverfly abundance	1500	int1	1,899.97	3.84	0.35/0.66	0.25
hoverfly abundance	1500	both	1,905.21	9.09	0.36/0.67	0.28
**hoverfly abundance**	**2000**	**non**	1,896.45	0.00	0.34/0.65	
hoverfly abundance	2000	int2	1,902.52	6.07	0.35/0.65	0.54
hoverfly abundance	2000	int1	1,899.91	3.46	0.35/0.66	0.22
hoverfly abundance	2000	both	1,905.11	8.67	0.36/0.67	0.25
**hoverfly abundance**	**2500**	**non**	1,896.86	0.00	0.33/0.65	
hoverfly abundance	2500	int2	1,902.96	6.10	0.34/0.66	0.54
hoverfly abundance	2500	int1	1,898.74	1.88	0.35/0.66	0.12
hoverfly abundance	2500	both	1,903.66	6.80	0.36/0.67	0.15
**hoverfly abundance**	**3000**	**non**	1,896.90	0.00	0.33/0.65	
hoverfly abundance	3000	int2	1,903.00	6.10	0.34/0.66	0.54
hoverfly abundance	3000	int1	1,898.44	1.54	0.35/0.67	0.10
hoverfly abundance	3000	both	1,903.19	6.29	0.36/0.68	0.13

***Table S5. Model comparison results for honey bee abundance** in relation to semi-natural habitat (SNH) cover at multiple spatial scales (250–3000 m). Candidate models included: baseline model without interactions (non), habitat × floral cover (int1), habitat × SNH (int2), and the full interaction model including both terms (both). Shown are small-sample corrected Akaike Information Criterion (AICc), differences from the best model (ΔAICc), marginal and conditional R^2 values, and p-values from likelihood ratio tests (LRT) comparing each interaction model to the baseline. The best-supported model per scale (smallest AICc) are in bold. Rows shaded in grey indicate the best supported model overall scales (AICc < 2 or significant LRT <0.05).*

Response	Predictor	Model	AICc	delta_AICc	R2 marg/cond	LRT_pval
**honey bee abundance**	**250**	**non**	1,943.22	0.00	0.39/0.52	
honey bee abundance	250	int2	1,946.21	3.00	0.41/0.53	0.18
honey bee abundance	250	int1	1,945.79	2.57	0.41/0.54	0.16
honey bee abundance	250	both	1,949.75	6.53	0.43/0.55	0.14
**honey bee abundance**	**500**	**non**	1,943.16	0.00	0.39/0.52	
honey bee abundance	500	int1	1,946.11	2.95	0.41/0.53	0.18
honey bee abundance	500	int2	1,948.60	5.44	0.40/0.53	0.44
honey bee abundance	500	both	1,952.80	9.64	0.42/0.54	0.33
**honey bee abundance**	**750**	**non**	1,942.96	0.00	0.40/0.52	
honey bee abundance	750	int1	1,945.91	2.95	0.41/0.53	0.18
honey bee abundance	750	int2	1,948.88	5.92	0.40/0.53	0.51
honey bee abundance	750	both	1,953.09	10.13	0.42/0.54	0.37
**honey bee abundance**	**1000**	**non**	1,943.08	0.00	0.39/0.52	
honey bee abundance	1000	int1	1,946.08	3.00	0.41/0.53	0.18
honey bee abundance	1000	int2	1,949.05	5.97	0.40/0.53	0.52
honey bee abundance	1000	both	1,953.41	10.33	0.42/0.53	0.39
**honey bee abundance**	**1500**	**non**	1,943.14	0.00	0.39/0.52	
honey bee abundance	1500	int1	1,946.10	2.97	0.41/0.53	0.18
honey bee abundance	1500	int2	1,949.37	6.23	0.40/0.53	0.56
honey bee abundance	1500	both	1,953.37	10.23	0.42/0.54	0.38
**honey bee abundance**	**2000**	**non**	1,942.79	0.00	0.40/0.52	
honey bee abundance	2000	int1	1,945.86	3.07	0.41/0.53	0.19
honey bee abundance	2000	int2	1,948.03	5.24	0.40/0.53	0.41
honey bee abundance	2000	both	1,952.39	9.60	0.42/0.54	0.32
**honey bee abundance**	**2500**	**non**	1,942.36	0.00	0.40/0.52	
honey bee abundance	2500	int1	1,945.45	3.09	0.42/0.53	0.19
honey bee abundance	2500	int2	1,948.46	6.09	0.40/0.53	0.54
honey bee abundance	2500	both	1,952.45	10.09	0.42/0.54	0.37
**honey bee abundance**	**3000**	**non**	1,942.64	0.00	0.40/0.52	
honey bee abundance	3000	int1	1,945.65	3.01	0.41/0.53	0.19
honey bee abundance	3000	int2	1,949.26	6.61	0.40/0.53	0.63
honey bee abundance	3000	both	1,953.04	10.40	0.42/0.53	0.39

***Table S6. Model comparison results for solitary bee richness** in relation to semi-natural habitat (SNH) cover at multiple spatial scales (250–3000 m). Candidate models included: baseline model without interactions (non), habitat × floral cover (int1), habitat × SNH (int2), and the full interaction model including both terms (both). Shown are small-sample corrected Akaike Information Criterion (AICc), differences from the best model (ΔAICc), marginal and conditional R^2 values, and p-values from likelihood ratio tests (LRT) comparing each interaction model to the baseline. The best-supported model per scale (smallest AICc) are in bold. Rows shaded in grey indicate the best supported model overall scales (AICc < 2 or significant LRT).*

Response	Predictor	Model	AICc	delta_AICc	R2 marg/cond	LRT_pval
**solitary bee richness**	**250**	**non**	**1,201.66**	**0.00**	**0.18/0.26**	
solitary bee richness	250	int1	1,203.14	1.48	0.22/0.28	0.10
solitary bee richness	250	int2	1,207.28	5.62	0.19/0.28	0.47
solitary bee richness	250	both	1,209.77	8.11	0.23/0.30	0.22
**solitary bee richness**	**500**	**non**	**1,200.76**	**0.00**	**0.18/0.25**	
solitary bee richness	500	int1	1,202.18	1.42	0.23/0.27	0.10
solitary bee richness	500	int2	1,207.23	6.46	0.19/0.27	0.60
solitary bee richness	500	both	1,208.95	8.18	0.24/0.28	0.22
**solitary bee richness**	**750**	**non**	**1,198.29**	**0.00**	**0.20/0.25**	
solitary bee richness	750	int1	1,199.60	1.31	0.24/0.27	0.10
solitary bee richness	750	int2	1,203.56	5.27	0.21/0.26	0.42
solitary bee richness	750	both	1,204.84	6.55	0.26/0.28	0.14
**solitary bee richness**	**1000**	**non**	**1,196.60**	**0.00**	**0.20/0.25**	
solitary bee richness	1000	int1	1,197.67	1.07	0.25/0.27	0.09
solitary bee richness	1000	int2	1,202.81	6.22	0.21/0.27	0.56
solitary bee richness	1000	both	1,204.15	7.56	0.26/0.29	0.19
**solitary bee richness**	**1500**	**non**	**1,194.61**	**0.00**	**0.21/0.26**	
solitary bee richness	1500	int1	1,195.53	0.92	0.26/0.28	0.08
solitary bee richness	1500	int2	1,198.53	3.93	0.23/0.28	0.26
solitary bee richness	1500	both	1,200.15	5.55	0.27/0.30	0.10
**solitary bee richness**	**2000**	**non**	**1,194.63**	**0.00**	**0.21/0.26**	
solitary bee richness	2000	int1	1,195.45	0.82	0.26/0.28	0.08
solitary bee richness	2000	int2	1,197.06	2.43	0.24/0.29	0.15
solitary bee richness	2000	both	1,198.66	4.04	0.28/0.31	0.06
**solitary bee richness**	**2500**	**non**	**1,193.39**	**0.00**	**0.22/0.26**	
solitary bee richness	2500	int2	1,194.22	0.83	0.26/0.28	0.08
solitary bee richness	2500	int1	1,193.69	0.30	0.25/0.30	0.06
**solitary bee richness**	**2500**	**both**	**1,195.11**	**1.72**	**0.29/0.32**	**0.03**
**solitary bee richness**	**3000**	**non**	**1,193.54**	**0.00**	**0.22/0.26**	
solitary bee richness	3000	int2	1,194.62	1.08	0.26/0.29	0.09
solitary bee richness	3000	int1	1,193.95	0.41	0.25/0.30	0.07
**solitary bee richness**	**3000**	**both**	**1,195.28**	**1.74**	**0.29/0.32**	**0.03**

***Table S7. Model comparison results for bumble bee richness** in relation to semi-natural habitat (SNH) cover at multiple spatial scales (250–3000 m). Candidate models included: baseline model without interactions (non), habitat × floral cover (int1), habitat × SNH (int2), and the full interaction model including both terms (both). Shown are small-sample corrected Akaike Information Criterion (AICc), differences from the best model (ΔAICc), marginal and conditional R^2 values, and p-values from likelihood ratio tests (LRT) comparing each interaction model to the baseline. The best-supported model per scale (smallest AICc) are in bold. Rows shaded in grey indicate the best supported model overall scales (AICc < 2 or significant LRT).*

Response	Predictor	Model	AICc	delta_AICc	R2 marg/cond	LRT_pval
**bumble bee richness**	**250**	non	653.97	0.00	0.08/0.12	
bumble bee richness	250	int2	662.32	8.35	0.08/0.12	0.93
bumble bee richness	250	int1	659.29	5.32	0.09/0.12	0.42
bumble bee richness	250	both	667.88	13.91	0.09/0.12	0.77
**bumble bee richness**	**500**	**non**	**654.18**	**0.00**	**0.08/0.12**	
bumble bee richness	500	int2	662.45	8.27	0.08/0.12	0.92
bumble bee richness	500	int1	657.97	3.79	0.09/0.13	0.25
bumble bee richness	500	both	666.46	12.28	0.09/0.13	0.59
**bumble bee richness**	**750**	**non**	**651.32**	**0.00**	**0.09/0.12**	
bumble bee richness	750	int2	659.73	8.41	0.09/0.12	0.94
bumble bee richness	750	int1	653.74	2.42	0.10/0.13	0.15
bumble bee richness	750	both	662.51	11.20	0.10/0.14	0.47
**bumble bee richness**	**1000**	**non**	**648.66**	**0.00**	**0.10/0.13**	
bumble bee richness	1000	int2	657.05	8.38	0.10/0.13	0.94
bumble bee richness	1000	int1	650.95	2.29	0.11/0.14	0.14
bumble bee richness	1000	both	659.72	11.06	0.11/0.14	0.46
**bumble bee richness**	**1500**	**non**	**645.60**	**0.00**	**0.10/0.13**	
bumble bee richness	1500	int2	654.17	8.57	0.10/0.13	0.96
bumble bee richness	1500	int1	648.18	2.58	0.11/0.14	0.16
bumble bee richness	1500	both	657.06	11.46	0.11/0.14	0.50
**bumble bee richness**	**2000**	**non**	**645.03**	**0.00**	**0.10/0.13**	
bumble bee richness	2000	int2	653.76	8.73	0.10/0.13	0.98
bumble bee richness	2000	int1	647.31	2.28	0.11/0.14	0.14
bumble bee richness	2000	both	656.25	11.22	0.11/0.14	0.47
**bumble bee richness**	**2500**	**non**	**649.01**	**0.00**	**0.09/0.13**	
bumble bee richness	2500	int2	657.78	8.77	0.09/0.13	0.98
bumble bee richness	2500	int1	654.10	5.08	0.10/0.13	0.39
bumble bee richness	2500	both	663.13	14.11	0.10/0.13	0.79
**bumble bee richness**	**3000**	**non**	**651.45**	**0.00**	**0.09/0.12**	
bumble bee richness	3000	int2	660.10	8.65	0.09/0.12	0.97
bumble bee richness	3000	int1	657.62	6.17	0.09/0.13	0.55
bumble bee richness	3000	both	666.62	15.17	0.09/0.13	0.89

***Table S8. Model comparison results for hoverfly richness** in relation to semi-natural habitat (SNH) cover at multiple spatial scales (250–3000 m). Candidate models included: baseline model without interactions (non), habitat × floral cover (int1), habitat × SNH (int2), and the full interaction model including both terms (both). Shown are small-sample corrected Akaike Information Criterion (AICc), differences from the best model (ΔAICc), marginal and conditional R^2 values, and p-values from likelihood ratio tests (LRT) comparing each interaction model to the baseline. The best-supported model per scale (smallest AICc) are in bold. Rows shaded in grey indicate the best supported model overall scales (AICc < 2 or significant LRT).*

Response	Predictor	Model	AICc	delta_AICc	R2 marg/cond	LRT_pval
**hoverfly richness**	**250**	**non**	**743.40**	**0.00**	**0.06/0.09**	
hoverfly richness	250	int2	751.21	7.81	0.06/0.09	0.85
hoverfly richness	250	int1	745.05	1.65	0.08/0.11	0.11
hoverfly richness	250	both	751.75	8.34	0.08/0.10	0.23
**hoverfly richness**	**500**	**non**	**745.67**	**0.00**	**0.06/0.09**	
hoverfly richness	500	int2	753.35	7.67	0.06/0.08	0.82
hoverfly richness	500	int1	752.23	6.56	0.06/0.09	0.62
hoverfly richness	500	both	759.99	14.31	0.07/0.09	0.81
**hoverfly richness**	**750**	**non**	**746.20**	**0.00**	**0.06/0.09**	
hoverfly richness	750	int1	753.79	7.59	0.06/0.08	0.81
hoverfly richness	750	int2	754.65	8.45	0.06/0.09	0.94
hoverfly richness	750	both	762.55	16.35	0.06/0.09	0.96
**hoverfly richness**	**1000**	**non**	**746.16**	**0.00**	**0.06/0.09**	
hoverfly richness	1000	int1	753.75	7.58	0.06/0.08	0.80
hoverfly richness	1000	int2	755.23	9.07	0.06/0.09	1.00
hoverfly richness	1000	both	763.18	17.02	0.06/0.08	0.99
**hoverfly richness**	**1500**	**non**	**746.17**	**0.00**	**0.06/0.09**	
hoverfly richness	1500	int1	753.75	7.58	0.06/0.08	0.80
hoverfly richness	1500	int2	754.63	8.46	0.06/0.09	0.95
hoverfly richness	1500	both	762.51	16.34	0.06/0.09	0.96
**hoverfly richness**	**2000**	**non**	**746.21**	**0.00**	**0.06/0.09**	
hoverfly richness	2000	int1	753.81	7.60	0.06/0.08	0.81
hoverfly richness	2000	int2	753.99	7.78	0.06/0.09	0.84
hoverfly richness	2000	both	761.75	15.54	0.06/0.09	0.92
**hoverfly richness**	**2500**	**non**	**746.17**	**0.00**	**0.06/0.09**	
hoverfly richness	2500	int2	753.82	7.64	0.06/0.08	0.82
hoverfly richness	2500	int1	752.80	6.63	0.06/0.09	0.63
hoverfly richness	2500	both	760.43	14.25	0.06/0.09	0.80
**hoverfly richness**	**3000**	**non**	**746.12**	**0.00**	**0.06/0.09**	
hoverfly richness	3000	int2	753.79	7.67	0.06/0.08	0.82
hoverfly richness	3000	int1	752.13	6.01	0.07/0.10	0.53
hoverfly richness	3000	both	759.75	13.63	0.07/0.09	0.74

Section S4: At maximum weak habitat-dependent effects on wild bee abundance and richness.

Likelihood ratio test–based inference indicated at best small, but detectable, improvements in model fit when habitat interactions with flower cover and the proportion of semi-natural habitat at large spatial scales were included. However, these improvements were not sufficient to outweigh the AIC penalty associated with increased model complexity. For transparency, we present the full results below:

Table S9. Anova summary output of GLMM *explaining the effects of habitat type, flower richness, flower cover, mean annual temperature and percentage of semi natural habitat (SNH) in the village surrounding at a large radius on pollinator abundance and richness for wild pollinators.*

response	predictors	χ^{2} [a]	df [b]	p.value	R^2 (marg)[c]	R^2 (cond)[d]
solitary bee abundance	habitat	6.36	4	0.174	0.39	0.49
	**flower richness**	23.44	1	<0.001***		
	**temperature**	**4.36**	**1**	**<0.05***		
	log(flower cover)	0.57	1	0.45		
	%SNH _3000m	8.84	1	0.07		
	**habitat:log(flower cover)**	**10.19**	**4**	**<0.05***		
	habitat:%SNH_3000m	8.84	4	0.07		
solitary bee richness	**habitat**	**11.77**	**4**	**<0.05***	0.29	0.32
	**flower richness**	**14.37**	**1**	**<0.001*** **		
	temperature	0.06	1	0.801		
	log(flower cover)	2.04	1	0.15		
	%SNH_radius_2500m	0.01	1	0.92		
	habitat:log(flower cover)	7.54	4	0.11		
	habitat:%SNH_3000m	8.08	4	0.089		

[a]Chi-square; [b]Degrees of freedom; [c]R^2 marginal; [d]R^2 conditional; Values in boldface indicate significant differences.

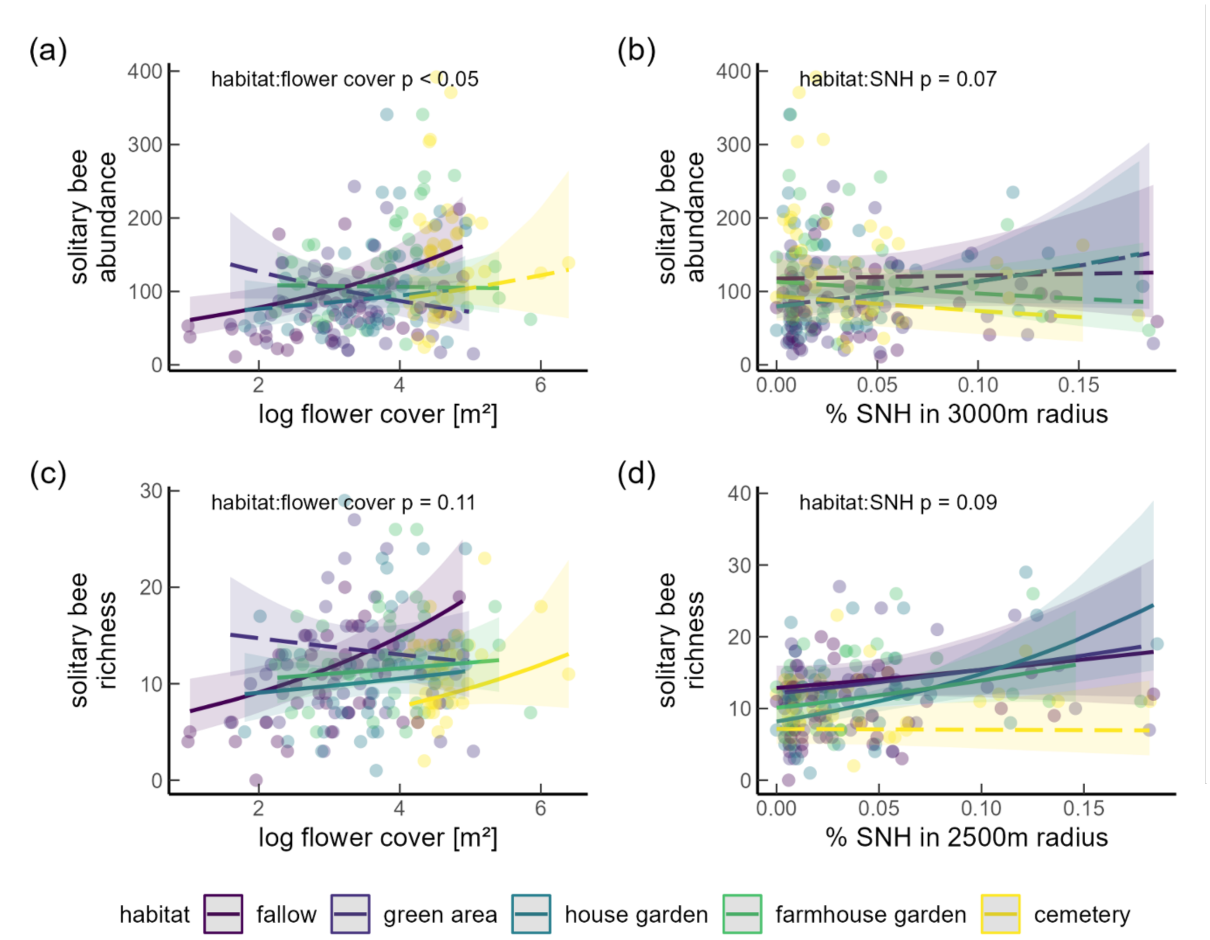

Figure S2. *Marginal habitat-dependent effects of flower cover, and the percentage of semi-natural habitats on the species abundance (a, b) and richness (c,d) of solitary bees. Flower cover is log-transformed. Shadows indicate the 95% confidence intervals. Solid lines indicate significant change with increasing percentage of SNH or flower cover.*

Our results suggest that wild bees benefited most strongly from flower cover increase in fallows and cemeteries. In contrast, a higher proportion of semi-natural habitat at larger spatial scales primarily enhanced wild bee richness in house gardens , to a lower extent in farmhouse gardens, green areas and fallows and not in cemeteries.

Section S5: Post-hoc comparison of habitat types on pollinator groups

***Table S10. Post-hoc comparisons of habitat types on pollinator abundance and richness**: The table presents estimated marginal means (Estimated Mean) for different habitat types and their corresponding standard errors (SE), confidence intervals (LCL and UCL), and statistical groupings based on Tukey's HSD test per factor. Habitat types with the same grouping symbol (e.g., "a", "b", "c") were not significantly different from each other (p > 0.05).*

Response	Habitat	Estimated Mean	SE	LCL	UCL	Letters
	cemetery	95.00	10.02	77.26	116.82	a
	fallow	106.50	10.80	87.29	129.92	a
solitary bee abundance	farmhouse garden	106.82	9.52	89.70	127.21	a
	green area	108.35	10.92	88.93	132.02	a
	house garden	93.06	8.13	78.41	110.45	a
	cemetery	25.82	3.30	20.11	33.17	a
	fallow	54.27	6.81	42.45	69.40	b
bumble bee abundance	farmhouse garden	50.93	5.46	41.27	62.85	b
	green area	45.30	5.54	35.65	57.56	b
	house garden	46.23	4.92	37.53	56.96	b
	cemetery	55.58	6.40	44.36	69.64	ab
	fallow	63.30	7.24	50.58	79.21	b
hoverfly abundance	farmhouse garden	50.49	5.13	41.37	61.63	ab
	green area	52.34	5.90	41.95	65.29	ab
	house garden	40.92	4.16	33.53	49.93	a
	cemetery	30.56	5.52	21.44	43.56	a
	fallow	50.39	9.05	35.43	71.66	ab
honey bee abundance	farmhouse garden	53.49	8.34	39.41	72.59	b
	green area	42.12	7.20	30.13	58.90	ab
	house garden	43.65	6.51	32.59	58.46	ab
	cemetery	8.25	0.71	6.97	9.77	a
	fallow	12.57	1.04	10.68	14.79	b
solitary bee richness	farmhouse garden	11.45	0.80	9.98	13.14	b
	green area	14.17	1.12	12.13	16.56	b
	house garden	10.51	0.75	9.15	12.08	ab
	cemetery	3.64	0.20	3.26	4.06	a
	fallow	5.03	0.26	4.54	5.57	c
bumble bee richness	farmhouse garden	4.51	0.20	4.13	4.92	bc
	green area	4.08	0.22	3.66	4.54	ab
	house garden	4.60	0.20	4.22	5.01	bc
	cemetery	3.65	0.29	3.13	4.26	a
	fallow	3.84	0.30	3.28	4.48	a
hoverfly richness	farmhouse garden	3.90	0.26	3.42	4.45	a
	green area	3.57	0.29	3.05	4.18	a
	house garden	4.06	0.26	3.57	4.61	a

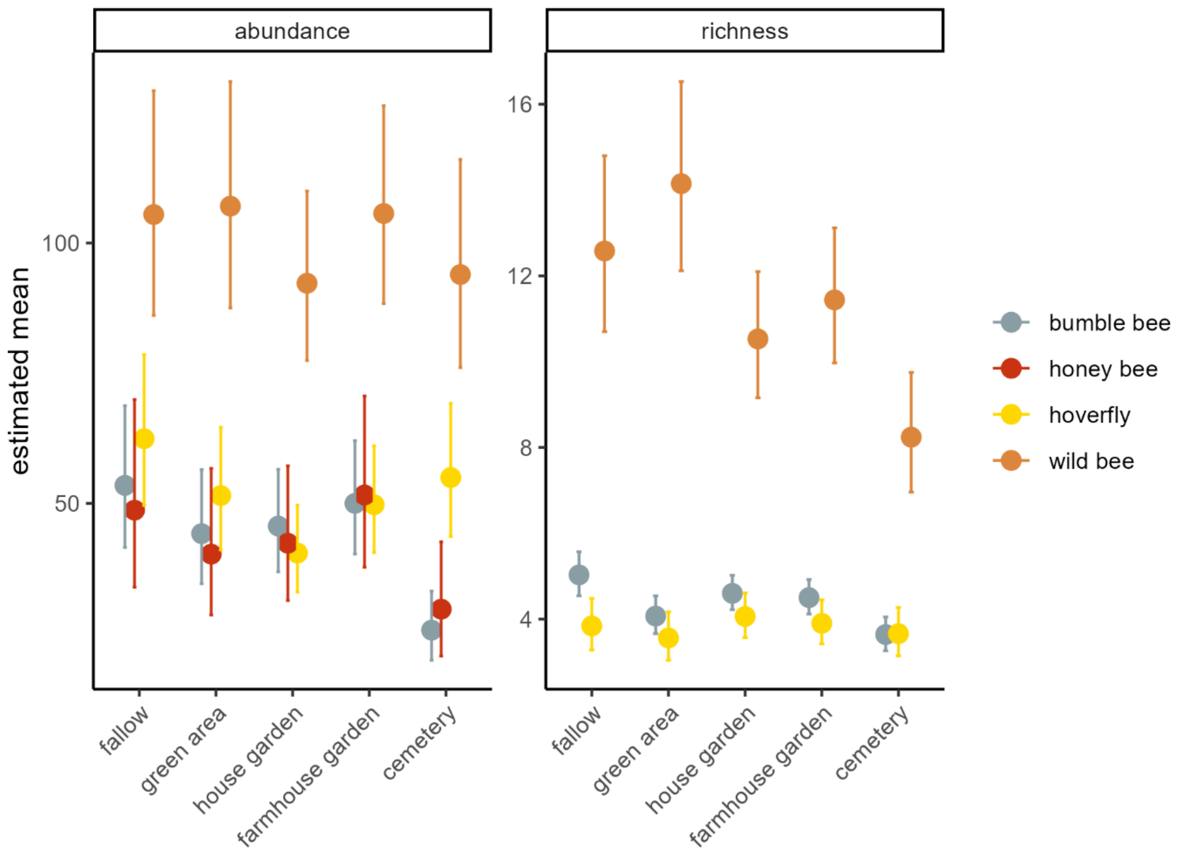

Figure S3. Effect of habitat types on pollinator groups. *Habitat types are ordered from left to right by increasing flower cover, richness, and proportion of ornamental plants.*

Section S6: Effects of ornamental cover on pollinator abundance and richness

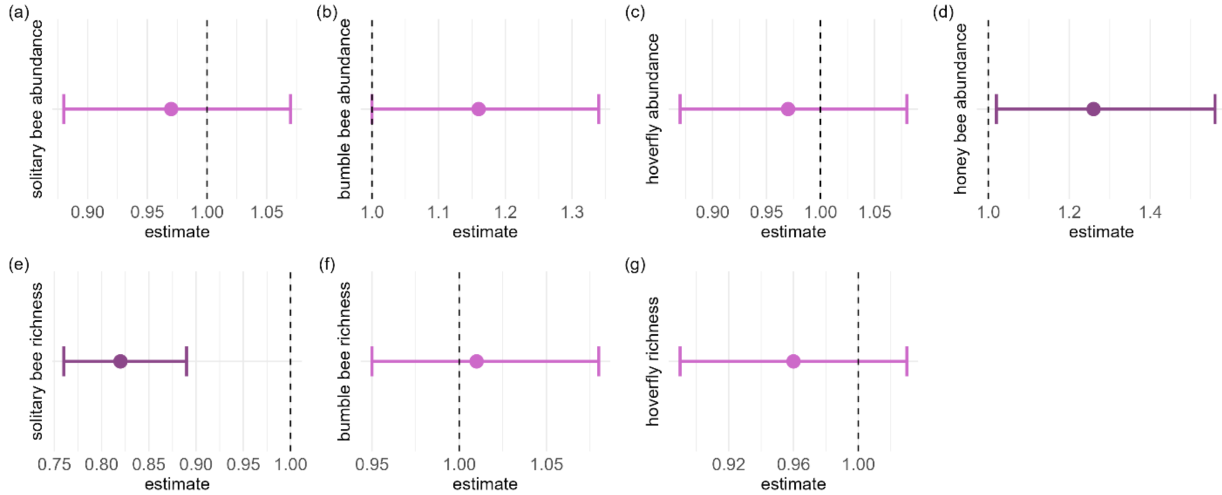

Figure S4. Coefficient estimates and 95% confidence intervals of glmmTMBs analysing the effect of the proportion of ornamental cover on pollinator species richness and abundance*, while controlling for total flower abundance (log transformed), total flower richness, temperature and the percentage of semi-natural habitat. Dark purple coefficient estimates and confidence intervals indicate significant results (p< 0.05). Parameter estimates have been back-transformed from log-link scale to the response scale, which is why the estimates change around 1 and not 0.*
